# Zero-profile anchored spacer versus conventional plate-cage construct in bilevel anterior cervical discectomy and fusion: a systematic review and meta-analysis

**DOI:** 10.1186/s13018-023-04134-4

**Published:** 2023-08-31

**Authors:** Yu Zhang, Jidong Ju, Jinchun Wu

**Affiliations:** Department of Orthopaedics, Jingjiang People’s Hospital, The Seventh Affiliated Hospital of Yangzhou University, Jingjiang 214500, Taizhou, Jiangsu Province China

**Keywords:** Zero-profile anchored spacers, Plate-cage constructs, Effectiveness and safety, Bilevel cervical spinal fusion, Meta-analysis

## Abstract

**Background:**

Zero-profile anchored spacers (ZAS) and plate-cage constructs (PCC) are currently employed when performing anterior cervical discectomy and fusion (ACDF). Nevertheless, the efficacy and safety of both devices in bilevel ACDF remain controversial. The goal of our meta-analysis is to assess the overall long-term efficacy and security among ZAS and PCC in bilevel ACDF.

**Methods:**

A search of four electronic databases was conducted to identify researches that compared ZAS with PCC for bilevel ACDF. Stata MP 17.0 software was used for this meta-analysis.

**Results:**

Nine researches with a total of 580 patients were involved. In comparison to PCC, ZAS significantly reduced intraoperative bleeding and postoperative dysphagia rates. No significant differences were found concerning operation time, JOA score, NDI score, cervical Cobb angle, fusion rates, the incidence of adjacent segmental degeneration (ASD) and implant sinking rates at last follow-up.

**Conclusion:**

Compared to PCC, ZAS achieved similar efficacy and security in bilevel ACDF with respect to operative time, JOA score, NDI score, cervical Cobb angle, fusion rates, implant sinking rates and ASD rates at final follow-up. It is worth noting that ZAS offered considerable benefits over conventional PCC for the reduction of intraoperative bleeding and postoperative dysphagia. Therefore, for patients requiring bilevel ACDF, ZAS seems superior to PCC. Given the limitations of our study, larger prospective randomised controlled trials are needed to establish reliable proof to consolidate our conclusions.

**Supplementary Information:**

The online version contains supplementary material available at 10.1186/s13018-023-04134-4.

## Introduction

As the population of the older generation grow, the frequency of degenerative diseases increases annually. Simultaneously, the population of patients affected by neck pain tends to rise every year given changing lifestyles and work-related stress. Neck pain occurring among adults is mostly blamed on cervical degenerative disc disease (CDDD), which may progress to spinal cord dysfunction in advanced stages [1, 2]. Several studies have explored effective ways to relieve spinal cord compression and restore spinal cord function in patients with CDDD. Commonly reported surgical treatments for cervical spinal conditions involve anterior approaches, posterior approaches, combined anterior and posterior approaches and various minimally invasive techniques [3–5].

In 1958, Smith and Robinson initially described anterior cervical surgery as a secure and useful technique in treating CDDD [6]. Anterior cervical discectomy and fusion (ACDF) is one of the most advanced cervical spine surgical methods and plays an important role in treating cervical spine disorders [7]. ACDF is usually performed with an interbody fusion cage, restoring disc height and avoiding implant migration with the application of an anterior plate. Whereas this plate-cage construct (PCC) has several advantages, such as immediate stabilisation, it also presents some drawbacks inherent to the plate. The most common disadvantages include fracture or loosening of the plate and screw, oesophageal interference and adjacent segment degeneration (ASD) and so on [8–10]. In addition, plate fixation requires additional manipulation and traction of the anterior cervical tissues, creating additional anterior volume, thus increasing the risk of dysphagia, especially in multisegmental ACDF [11].

In an effort to reduce the incidence of plate-related complications, one novel zero-profile anchored spacer (ZAS) was developed, which allowed the insertion of self-locking screws into the adjacent vertebral body by passing across the fusion construct rather than utilizing an anterior plate [12]. The crucial distinction between ZAS and PCC is avoiding the necessity for exposing a great deal of anatomical region during the procedure, thereby considerably reducing operative trauma and postsurgical scarring. Furthermore, ZAS reduces the occurrence of associated postoperative complications involving haematoma, adjacent segment degeneration and dysphagia without invading vital structures in the anterior cervical spine [13, 14]. However, given the limited published data, there is still no consensus among spine surgeons on the best technique to achieve solid fusion and improve clinical outcomes in ACDF.

Meta-analyses were conducted to further evaluate the efficacy of ZAS and PCC for the treatment of CDDD, but the results remained controversial [15–20]. Previous meta-analyses showed that ZAS had considerable superiority to PCC in decreasing surgical duration, intraoperative bleeding and postoperative dysphagia and ASD rates at long-term follow-up [19]. However, one study regarding multisegmental ACDF found that ZAS decreased the incidence of postsurgical dysphagia, whereas it was inferior to PCC at maintaining the cervical Cobb angle after surgery [20]. Bilevel CDDD is a specific type of CDDD presenting with a more complex pathology, including continuous and noncontinuous CDDD, which severely affects patients' quality of life [21–23]. Currently, there are few meta-analyses comparing the outcomes of ZAS versus PCC in bilevel ACDF. Hence, the goal of this review is to investigate the efficacy and safety of ZAS in bilevel ACDF compared with conventional PCC, thereby providing clinicians with compelling proof to support their therapeutic judgement.

## Methods

### Literature search

The literature search was carried out by means of the following electronic databases: PubMed, Cochrane Library, Embase and Web of Science. The publication date was from the creation of the database to 1 June, 2023. The full query string of the individual databases was documented in Additional file 1. Inclusion was initially assessed on the basis of the title and abstract of the studies. Thereafter, trials were selected for inclusion following full-text review. If several trials reported findings obtained from the same data source, the latest trial was considered for inclusion. In addition, references to papers were screened to determine any relevant trials missing from the primary literature screening. The selective procedure was completed by two separate assessors. Consensus was reached between the two assessors for final inclusion. The papers were cross-referenced to identify potential related trials. In cases of uncertainties, discussions were held with the third author and a decision was made pending the definitive result.

### Selection criteria

Inclusion criteria: (1) Trial type: randomised controlled trial (RCT) or observational study (OS) which analysed the results of ZAS with traditional PCC in bilevel ACDF. ZAS was considered as treatment group and PCC as control group. (2) Patients with clear clinical and radiological diagnosis of CDDD requiring bilevel ACDF. (3) Trials reported one or more of the below endpoints: operative duration, intraoperative blood loss (IBL), Japanese Orthopaedic Association (JOA) scores, cervical Cobb angle, fusion rates, Neck Disability Index (NDI) scores, cage subsidence rates, postoperative dysphagia rates, ASD rates. (4) Researches with a follow-up period of at least 1 year after the operation.

Exclusion criteria: 1. Studies involving people with single level or multiple levels (> 2 levels) of CDDD or other cervical spinal disorders (e.g., fractures, infections, tumours and congenital deformities) or with records of operations on the cervical spine. 2. Trials without comparisons. 3. Repeated reports, reviews, meta-analyses, case reports, biomechanical researches, cadaveric studies, animal studies, letters and conference abstracts. 4. Raw information that was not complete or available for analyses. 5. Literature not written in English.

### Extraction of data

Using a standardised form, two assessors extracted information independently. Any discrepancies in appraisal were reconciled through consultation to a third investigator. In the selected publications, information was extracted as follows: primary investigator, published date, research design and nation, type of bilevel ACDF (continuous or noncontinuous), number of participants, ages, follow-up duration and outcomes.

### Quality assessment

Two investigators were appointed to evaluate the quality of the eligible trials individually. The Newcastle–Ottawa Scale (NOS) was applied to evaluate observational studies, and high quality was described as being scoring between six and nine. Besides, the potential risk of bias for RCTs was investigated by applying the Cochrane Collaboration's risk of bias tool. The results were subsequently checked against each other and, where necessary, a third assessor was invited to ensure agreement.

### Statistical methodology

For the meta-analyses, Stata MP 17.0 (Stata Corporation, College Station, TX, USA) was utilised. We calculated effect sizes as odds ratios (OR) and 95% confidence intervals (CI) for all binary endpoints. Regarding continuous endpoints, effect sizes were defined as weighted mean differences (WMD) and 95% CIs. First of all, we tested the heterogeneity of the eligible trials by calculating the I^2^ statistic and the Cochrane Q test. Where *I*^2^ > 50% or *p*-value < 0.05 demonstrated heterogeneity between included trials, a random-effect model was applied. If not, a fixed-effect model was employed for pooling. Subsequently, different types of bilevel ACDF were considered as a possible source of heterogeneity. Subgroup analyses were undertaken to further investigate possible causes of heterogeneity. Furthermore, sensitivity analyses were carried out by removing each individual trial stepwise to determine the robustness of the combined findings. Ultimately, the risk of publication bias was tested by means of Egger's test. In our study, *p*-value < 0.05 was adopted to indicate statistical significance.

## Results

### Search process and results

**Fig. 1:**
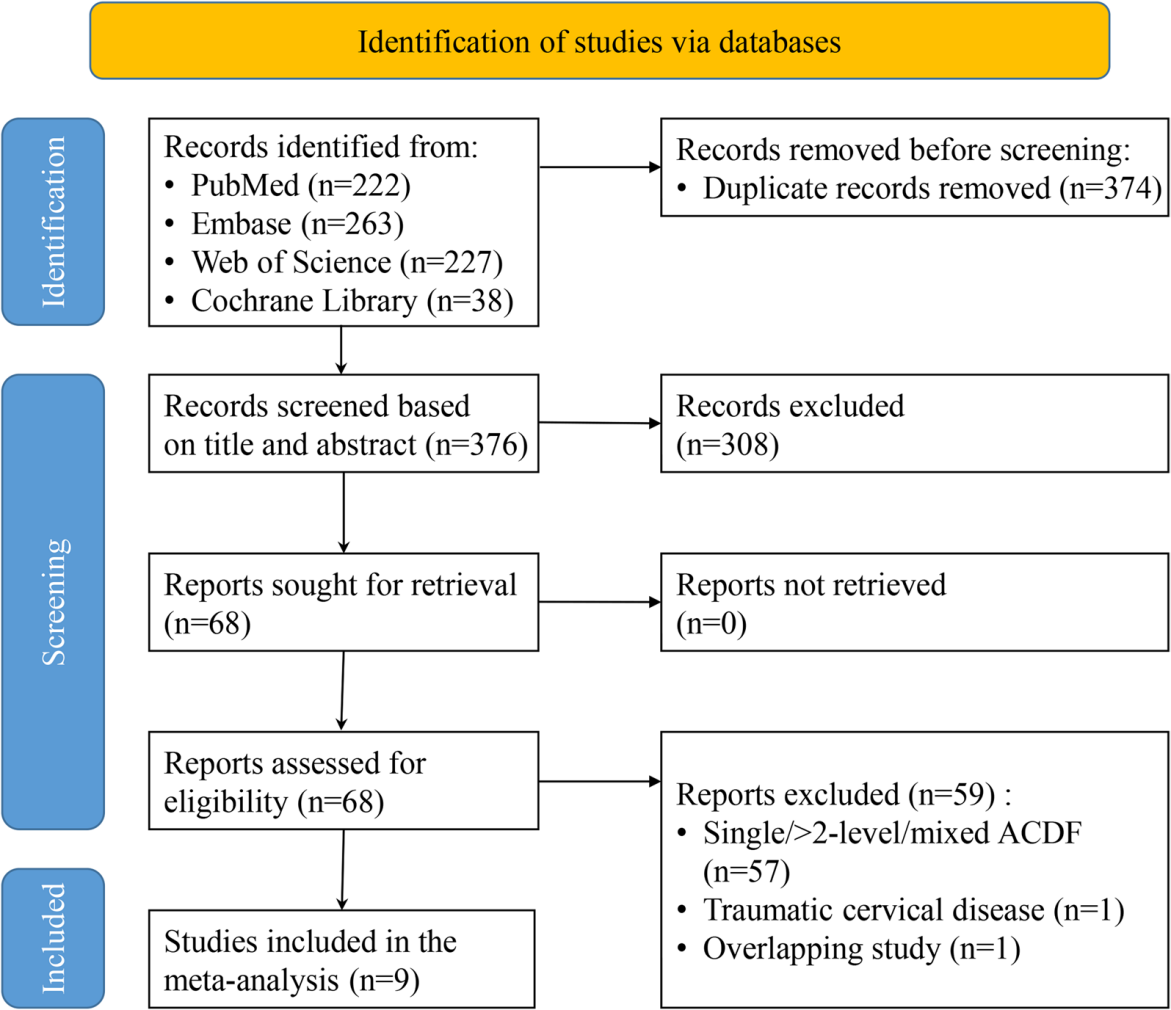
Flow chart of literature search

Originally, 750 related papers were chosen via a thorough retrieval of the online database. Subsequently, 374 duplicated articles were discarded. Afterwards, 308 articles that were not eligible for inclusion were deleted according to the title and abstract scanning process. Lastly, following reading the full text of the remaining 68 papers, nine were eventually involved in this meta-analysis [23–31] (Fig. 1).

### Study characteristics

Two RCTs and seven observational studies were eligible. A total of 580 participants received bilevel ACDF between 2015 and 2022 (Table 1). Of these, six studies involved continuous bilevel ACDF, while two papers involved noncontinuous bilevel ACDF and the remaining one did not mention a specific bilevel type.

**Table 1:** Study characteristics

Author	Publication date	Country	Study design	Type of bilevel	Sample size		Age (years)		Follow-up (months)	
					ZAS	PCC	ZAS	PCC	ZAS	PCC
Lu	2018	China	OS	Noncontiguous	22	24	56.6 ± 6.4	58.6 ± 7.2	30.5 ± 5.2	32.1 ± 6.5
Shi	2016	China	OS	Noncontiguous	34	31	56.1 ± 4.5	55.8 ± 4.9	> 24	> 24
Thind	2022	USA	OS	Contiguous	43	41	54.8 ± 8.8	58.1 ± 11.5	14.1 ± 6.2	20.4 ± 10.5
He	2022	China	OS	Contiguous	35	34	61.59 ± 8.21	60.15 ± 7.52	26.6 ± 3.4	27.1 ± 3.8
Scholz	2020	Germany	RCT	Contiguous	21	20	58	58	24	24
Yun	2016	Korea	OS	Contiguous	31	32	53.29 ± 7.55	54.18 ± 9.87	12.77 ± 7.85	13.62 ± 9.21
Zavras	2022	USA	RCT	Contiguous	10	10	53.50 ± 10.82	62.08 ± 9.17	12	12
Chen	2015	China	OS	Contiguous	37	32	48.9 ± 4.0	49.5 ± 4.2	40.6 ± 9.2	43.5 ± 10.4
Yang	2016	China	OS	Unspecified	60	63	47.90 ± 8.84	48.03 ± 8.46	36	36

*ZAS* zero-profile anchored spacer; *OS* observational study; *PCC* plate-cage construct; *RCT* randomised controlled trial

### Quality assessment

We evaluated the quality of observational trials according to NOS scores, and then details of the quality evaluation are presented with Table 2. The eventual findings revealed that every publication received a score of at least 7 points and was classified as high quality.

**Table 2:** Quality evaluation of observational trials using Newcastle–Ottawa Scale

Study	Selection	Comparability	Outcome	Total score
Lu 2018	3	1	3	7
Shi 2016	4	1	3	8
Thind 2022	4	1	3	8
He 2022	4	1	3	8
Yun 2016	4	1	3	8
Chen 2015	4	1	3	8
Yang 2016	4	1	3	8

**Fig. 2:**
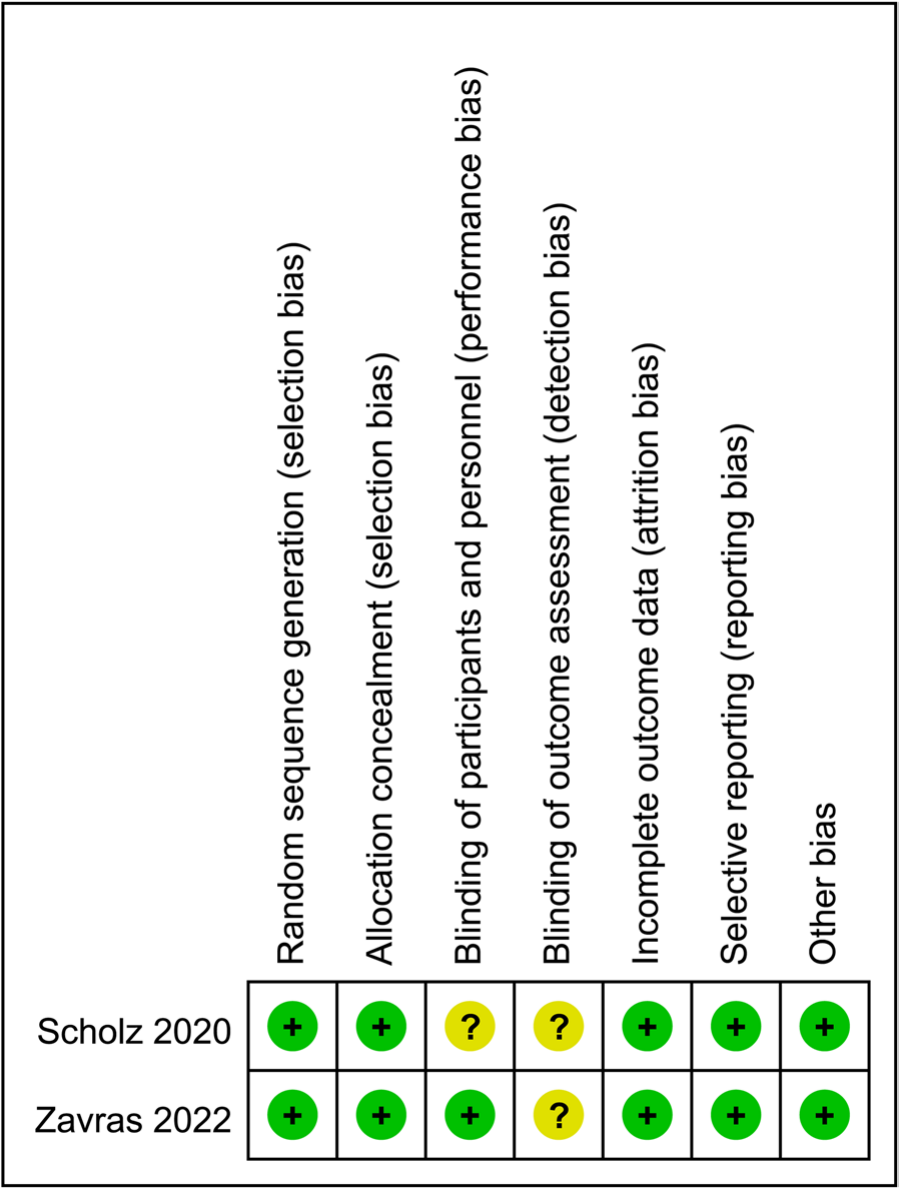
Quality appraisal of randomised controlled trials

Figure 2 presents the results of the quality appraisal of 2 RCTs by use of the Cochrane Collaboration's risk of bias tool. Two studies were not sufficiently blinded to patients, physicians or measurers, and we therefore considered implementation bias and measurement bias to be unclear.

## Results of meta-analyses

### Intraoperative bleeding

**Fig. 3:**
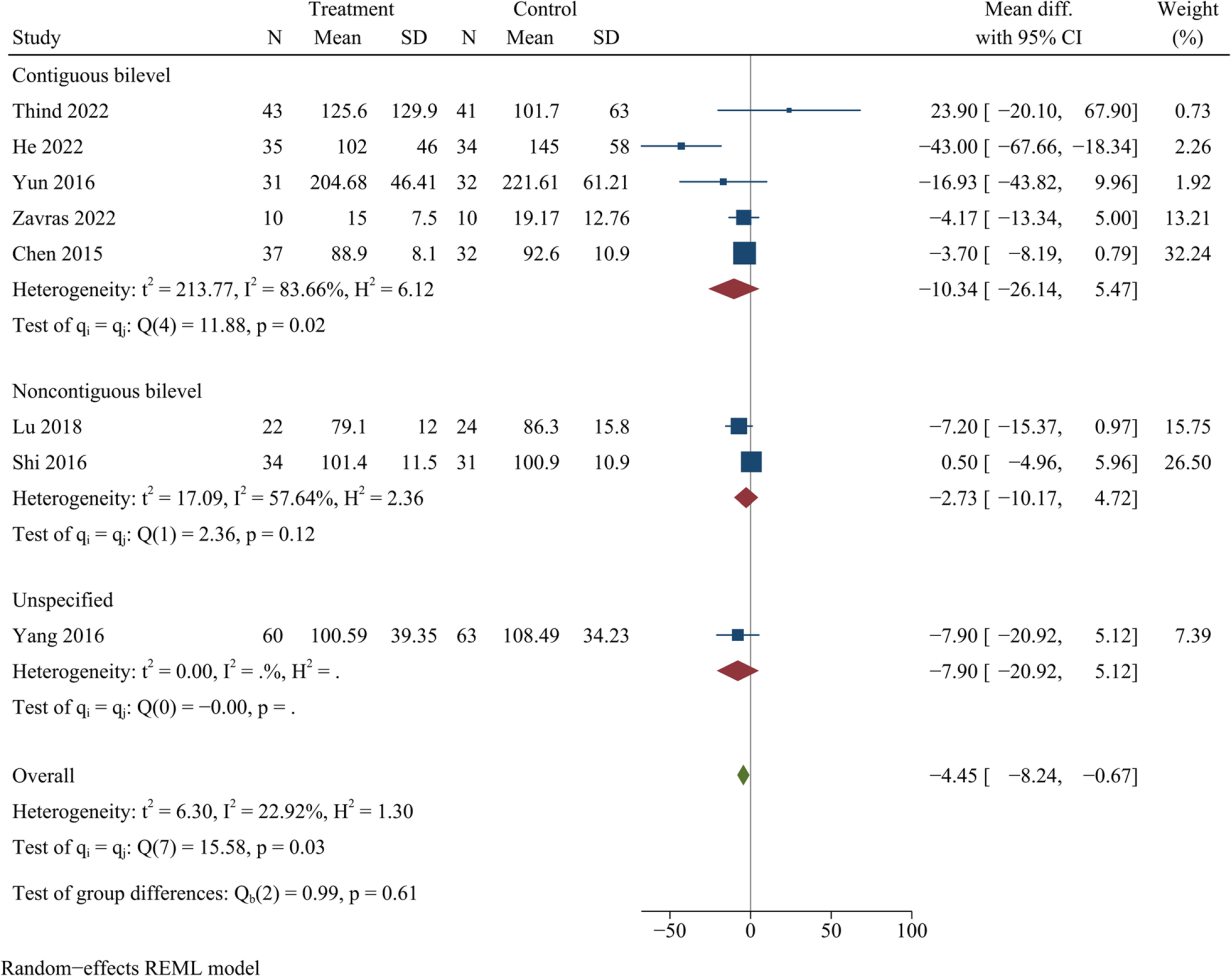
Forest plot of intraoperative blood loss

Eight publications reported intraoperative bleeding, including 272 cases at ZAS group and 267 cases at PCC group. IBL was less at ZAS group in comparison with PCC group, showing statistically significant difference [WMD =  − 4.45, 95% CI ( − 8.24,  − 0.67), *p* = 0.02] (Table 3). On further subgroup analysis, there were no significant differences in intraoperative bleeding among ZAS and PCC groups for either continuous or noncontinuous ACDF (Fig. 3).

**Table 3:** Findings of meta-analysis

Outcome	Study	Effect size	95% CI		*P*-value	Heterogeneity		Effect	Egger’s test
	size	WMD/OR	Lower limit	Upper limit		*I*^2^ (%)	*p*-Value	Model	*p*-Value
Intraoperative blood loss	8	− 4.45	− 8.24	− 0.67	0.02	22.92	0.03	Random	0.08
Operation time	8	− 3.44	− 15.62	8.74	0.58	94.98	< 0.01	Random	0.35
JOA score	5	− 0.02	− 0.28	0.25	0.91	0.00	0.83	Fixed	0.54
NDI score	5	0.06	− 0.38	0.50	0.79	0.00	0.99	Fixed	0.67
Cervical Cobb angle	5	− 0.66	− 1.68	0.35	0.20	0.00	0.47	Fixed	0.21
Fusion rate	9	0.75	0.41	1.39	0.36	0.00	0.99	Fixed	0.69
Cage subsidence	5	1.38	0.59	3.22	0.46	0.00	0.83	Fixed	0.52
Adjacent segment degeneration	5	0.61	0.29	1.28	0.19	0.00	0.89	Fixed	0.54
Dysphagia	7	0.53	0.36	0.79	< 0.01	9.70	0.35	Fixed	0.60

*NDI* neck disability index; *JOA* Japanese orthopaedic association; *WMD* weighted mean difference; *OR* odds ratio; *CI* confidence interval

### Surgical duration

**Fig. 4:**
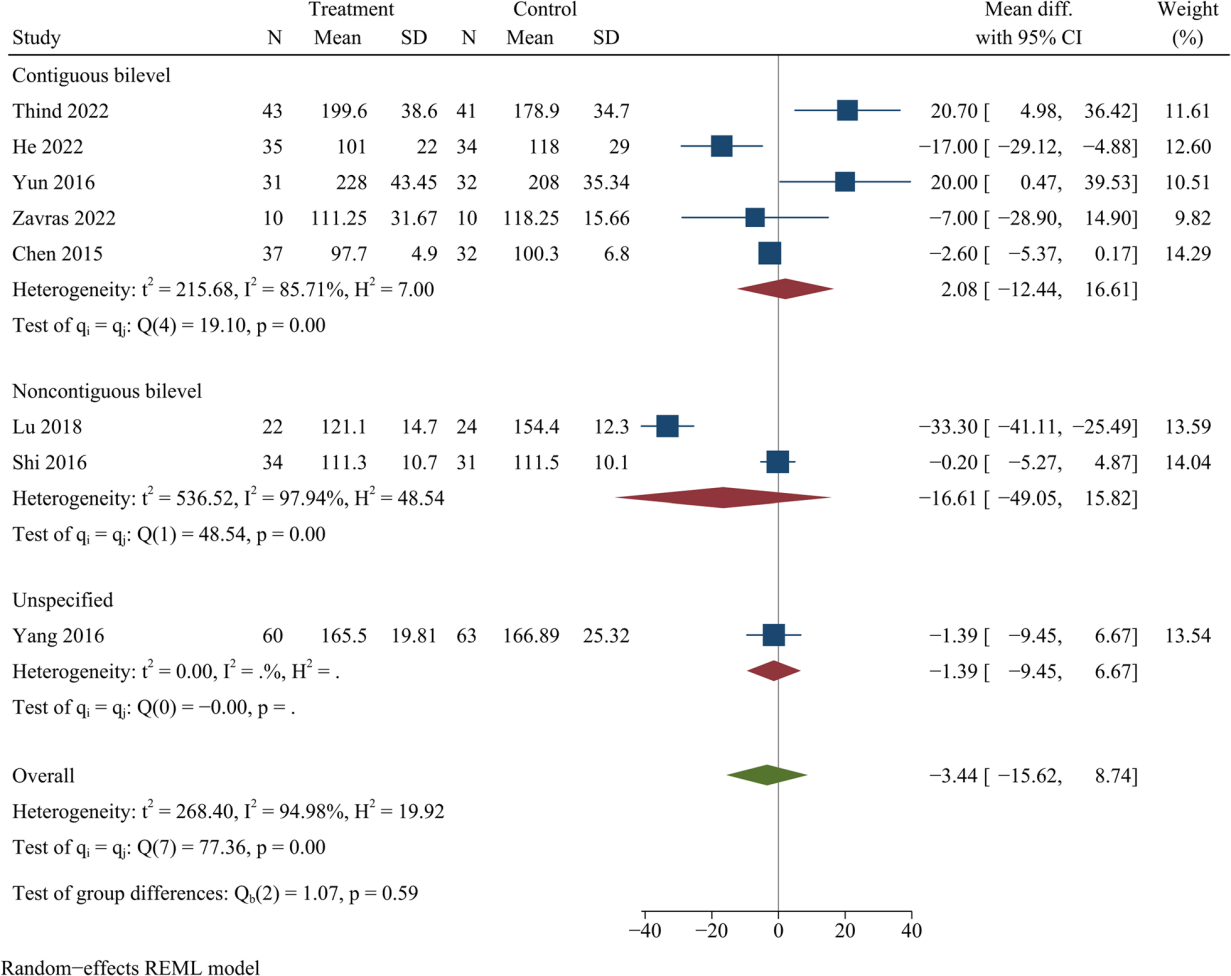
Forest plot of operation time

Eight trials examined operative duration involving a sample size of 539 participants. The operative time in ZAS group did not differ significantly from that in PCC group [WMD =  − 3.44, 95% CI ( − 15.62, 8.74), *p* = 0.58]. On further subgroup analysis, there was no significant difference in operative time between ZAS and PCC groups regarding either continuous or noncontinuous ACDF (Fig. 4).

### JOA score

**Fig. 5:**
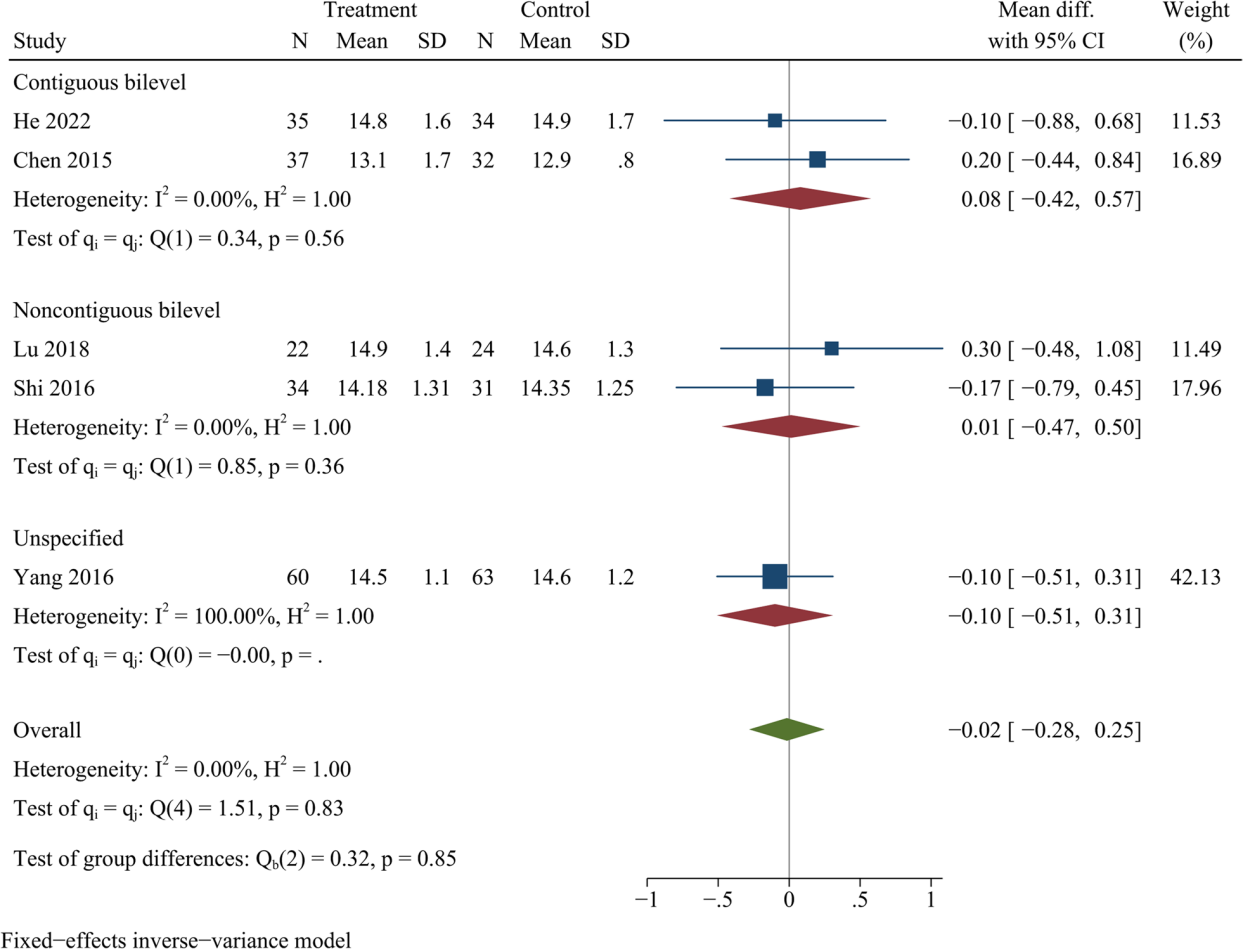
Forest plot of final JOA score

Five trials with a total of 372 participants were pooled for JOA score at the end of follow-up. Overall, the analysis indicated there were no significant differences in JOA scores among ZAS and PCC at final follow-up [WMD =  − 0.02, 95% CI ( − 0.28, 0.25), p = 0.91]. Following further subgroup analysis, there were no significant differences in JOA score between ZAS and PCC groups in both continuous and noncontinuous ACDF (Fig. 5).

### NDI score

**Fig. 6:**
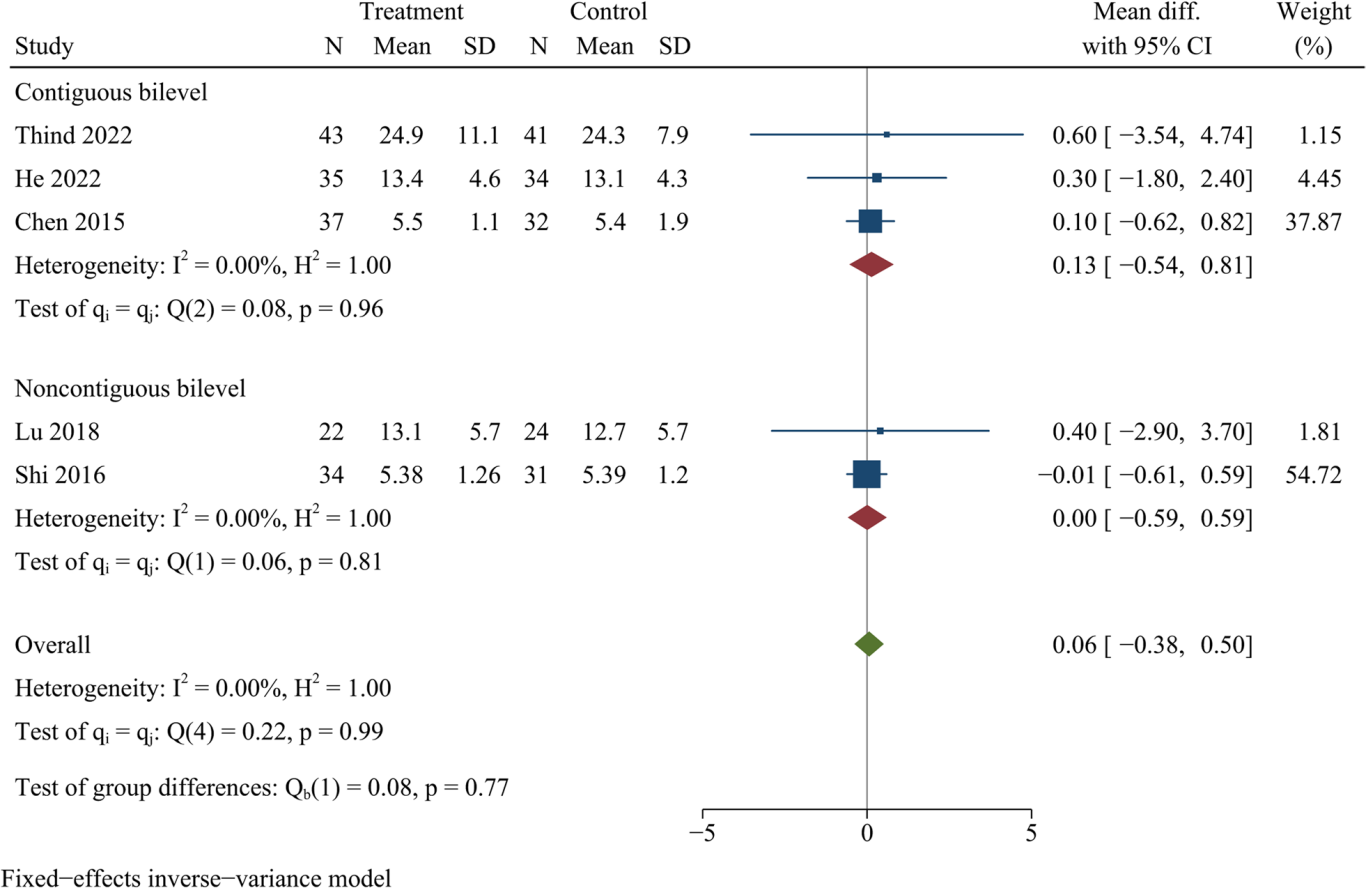
Forest plot of final NDI score

Five publications reported the final NDI score, including a total of 333 participants (171 in ZAS group and 162 in PCC group). The difference in NDI scores between both groups at last follow-up was not statistically significant [WMD = 0.06, 95% CI ( − 0.38, 0.50), *p* = 0.79]. On further subgroup analysis, there were no significant differences in NDI scores between ZAS and PCC groups for either continuous or noncontinuous ACDF (Fig. 6).

### Cervical Cobb angle

**Fig. 7:**
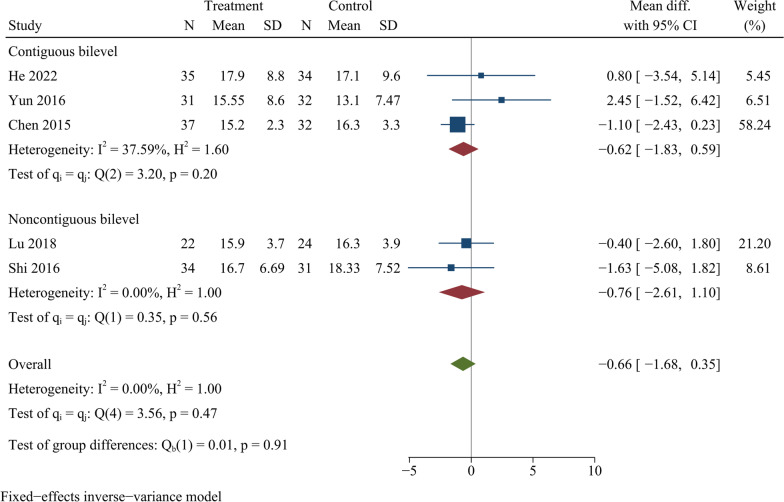
Forest plot of cervical curvature

Five publications covering 312 cases recorded the cervical Cobb angle at the last follow-up. There was no statistically significant difference in the comparison of cervical Cobb angle at last follow-up between ZAS and PCC groups [WMD =  − 0.66, 95% CI ( − 1.68, 0.35), *p* = 0.20]. On further subgroup analysis, there were no significant differences in cervical Cobb angle between ZAS and PCC groups in either continuous or noncontinuous ACDF (Fig. 7).

### Fusion rate

**Fig. 8:**
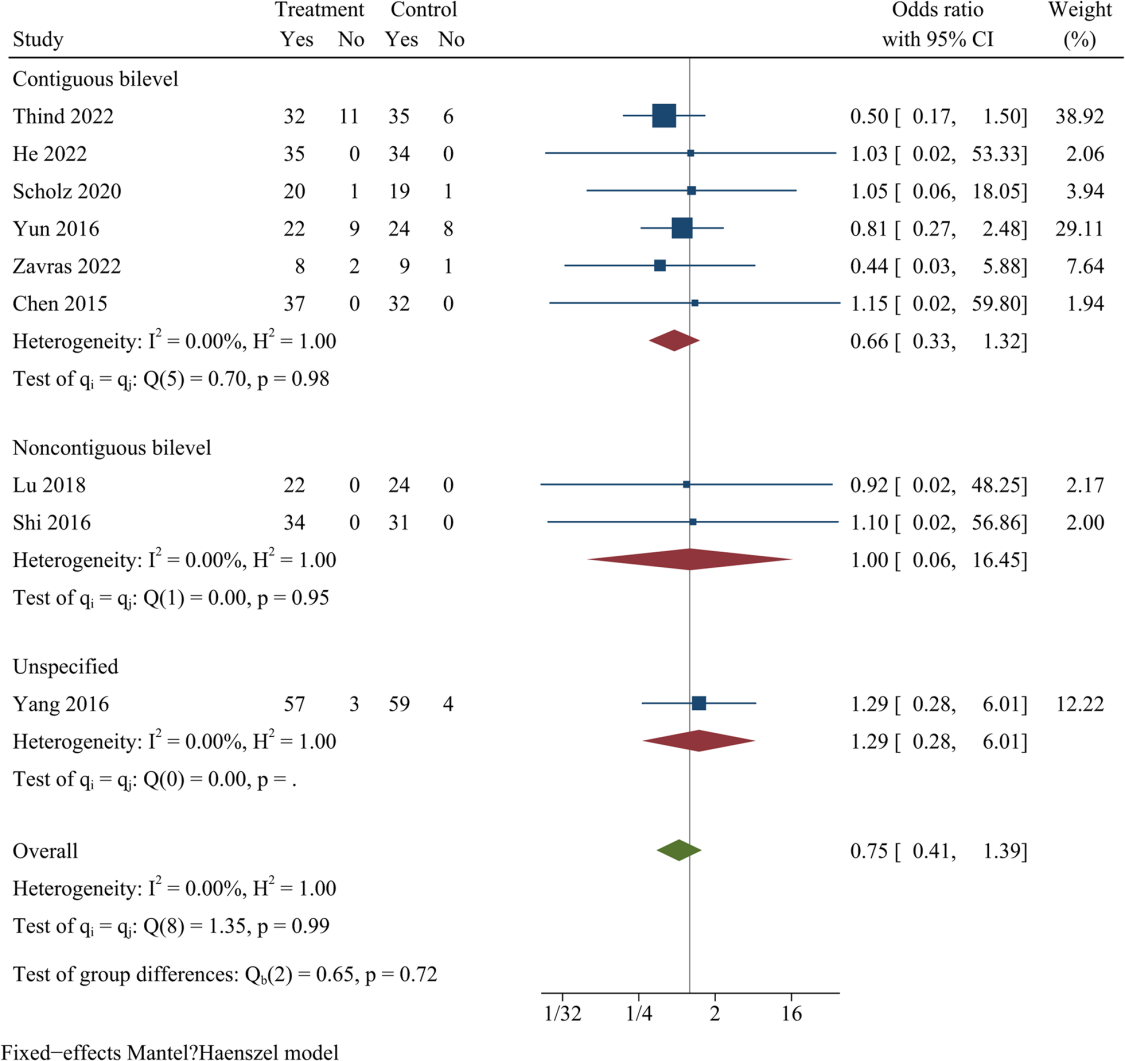
Forest plot of fusion rate

Nine studies reported the fusion rate at final follow-up, and included a total of 580 cases. At final follow-up, the difference in fusion rate between ZAS and PCC groups was not statistically significant [OR = 0.75, 95% CI (0.41, 1.39), *p* = 0.36]. On further subgroup analysis, there were no significant differences in fusion rate between ZAS and PCC groups in either continuous or noncontinuous ACDF (Fig. 8).

### Cage subsidence

**Fig. 9:**
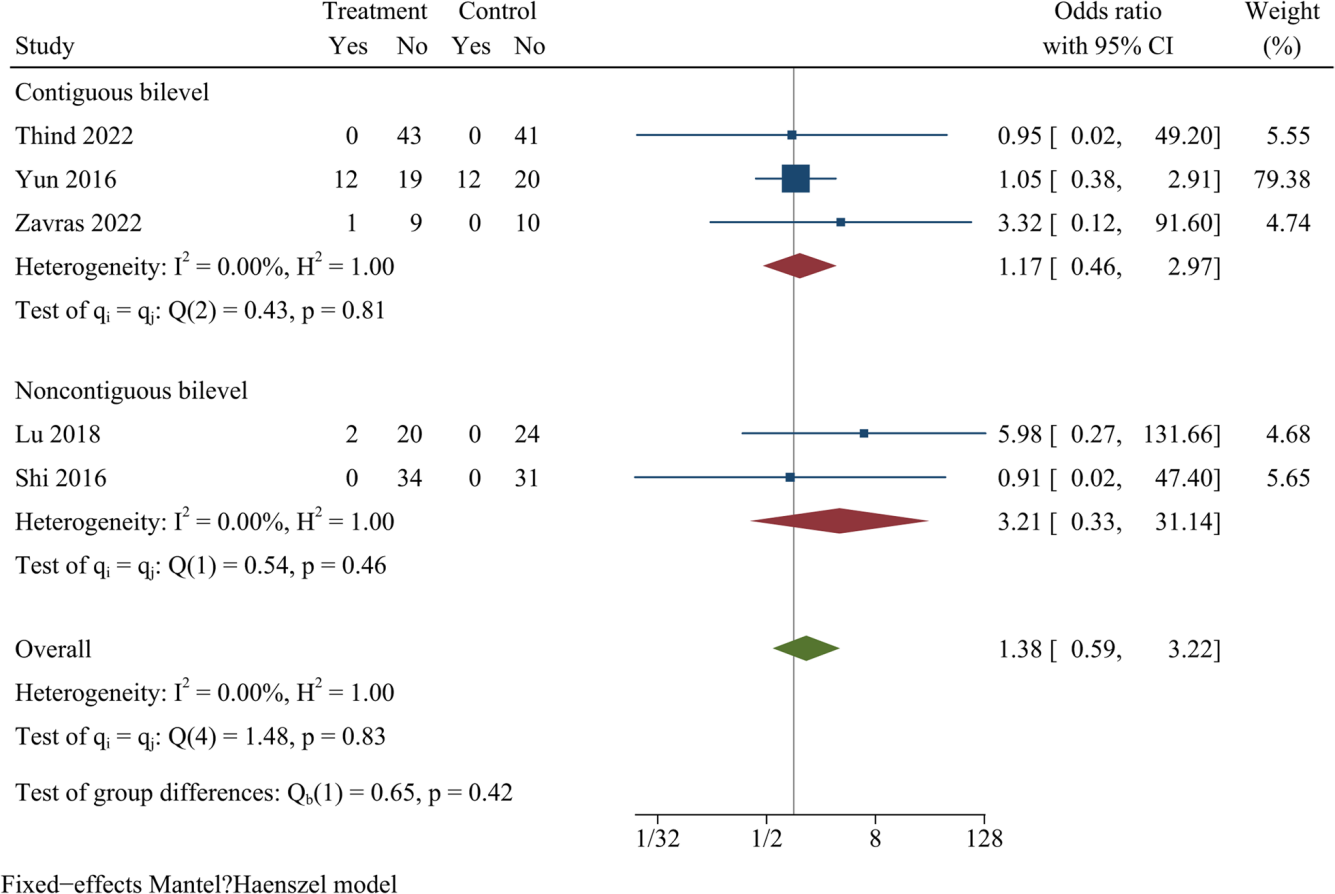
Forest plot of cage sinking rate

A total of 278 participants were examined in five papers reporting information about the rate of cage subsidence. The findings demonstrated no statistically significant differences in cage sinking rate between the two groups [OR = 1.38, 95% CI (0.59, 3.22), *p* = 0.46]. On further subgroup analysis, there was no significant difference in cage sinking rates between ZAS and PCC groups in both continuous and noncontinuous ACDF (Fig. 9).

### Adjacent segmental degeneration

**Fig. 10:**
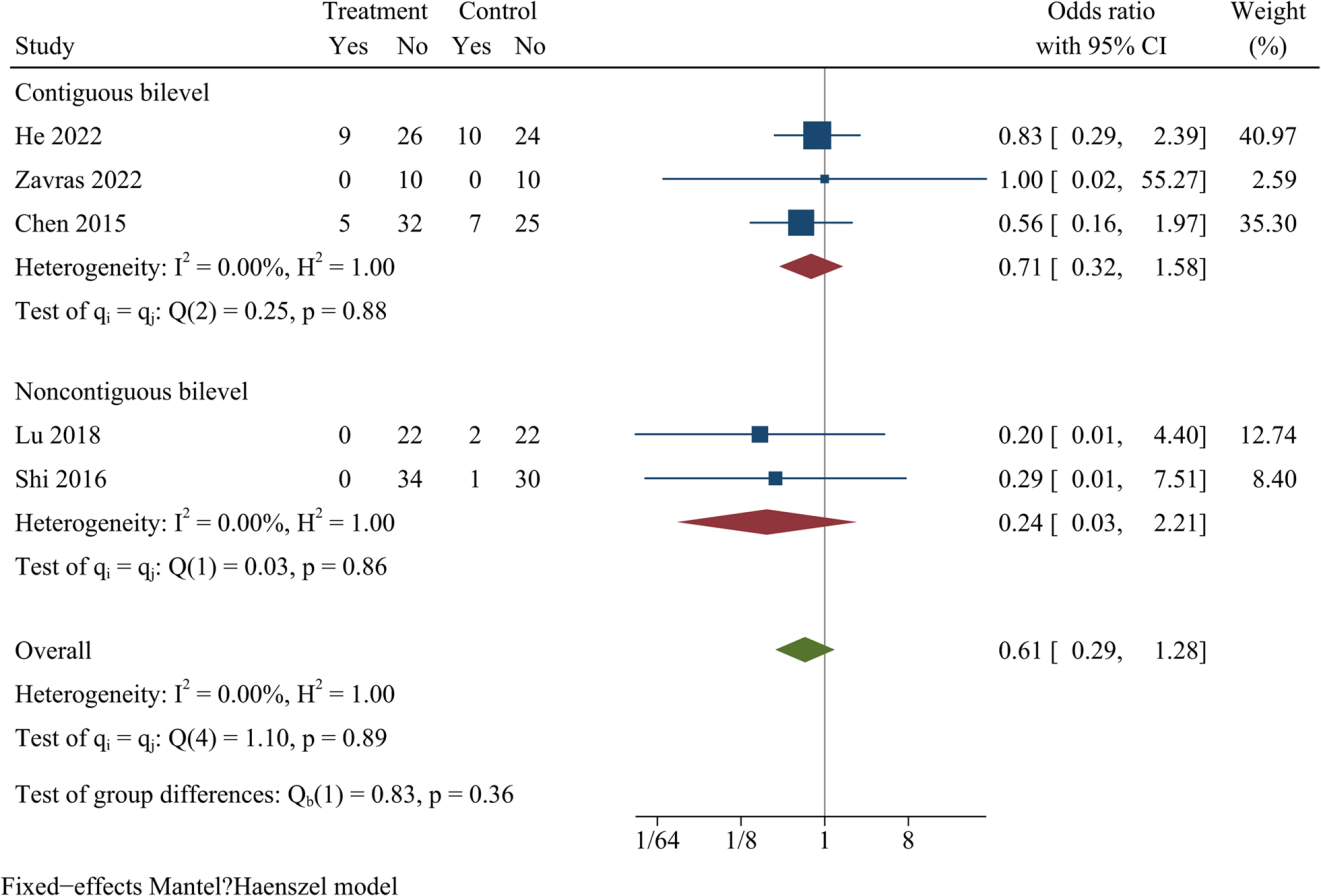
Forest plot of adjacent segmental degeneration rates

Five publications reported the ASD rate at the last follow-up. Overall, the analysis demonstrated no statistically significant difference in ASD rate between ZAS and PCC groups [OR = 0.61, 95% CI (0.29, 1.28), *p* = 0.19] (Fig. 10). Further subgroup analysis showed no significant difference in ASD rates between ZAS and PCC groups in both continuous and noncontinuous ACDF.

### Dysphagia

**Fig. 11:**
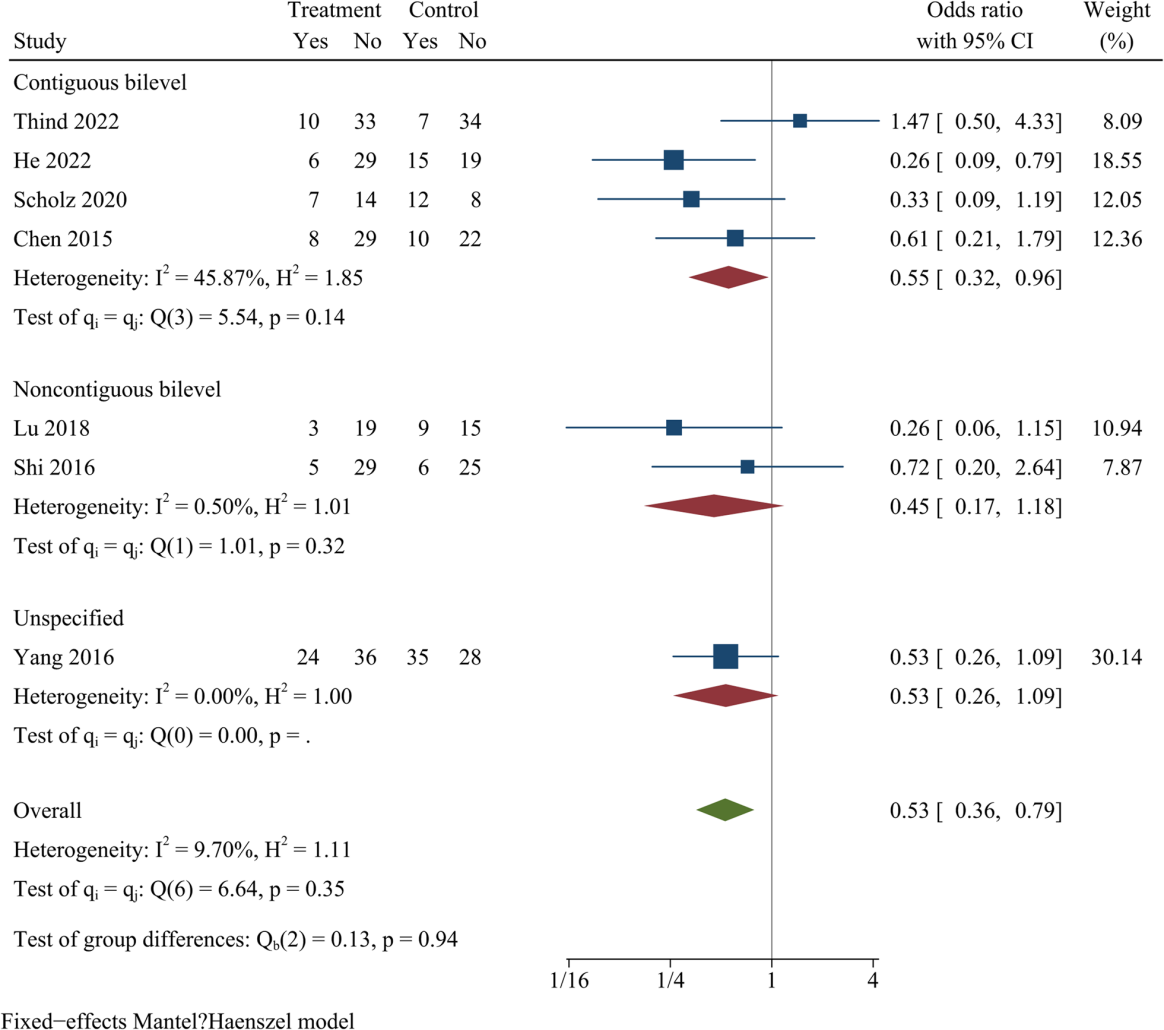
Forest plot of postoperative dysphagia rates

The incidence of postoperative dysphagia was reported in seven studies. The findings showed a statistically significant difference in the postoperative dysphagia rate between ZAS and PCC groups [OR = 0.53, 95% CI (0.36, 0.79), *p* < 0.01]. On further subgroup analysis, the postoperative dysphagia rate was significantly smaller in ZAS group than in PCC group in continuous ACDF. However, in noncontinuous ACDF, there was no significant difference between two groups (Fig. 11).

### Sensitivity analysis

**Fig. 12:**
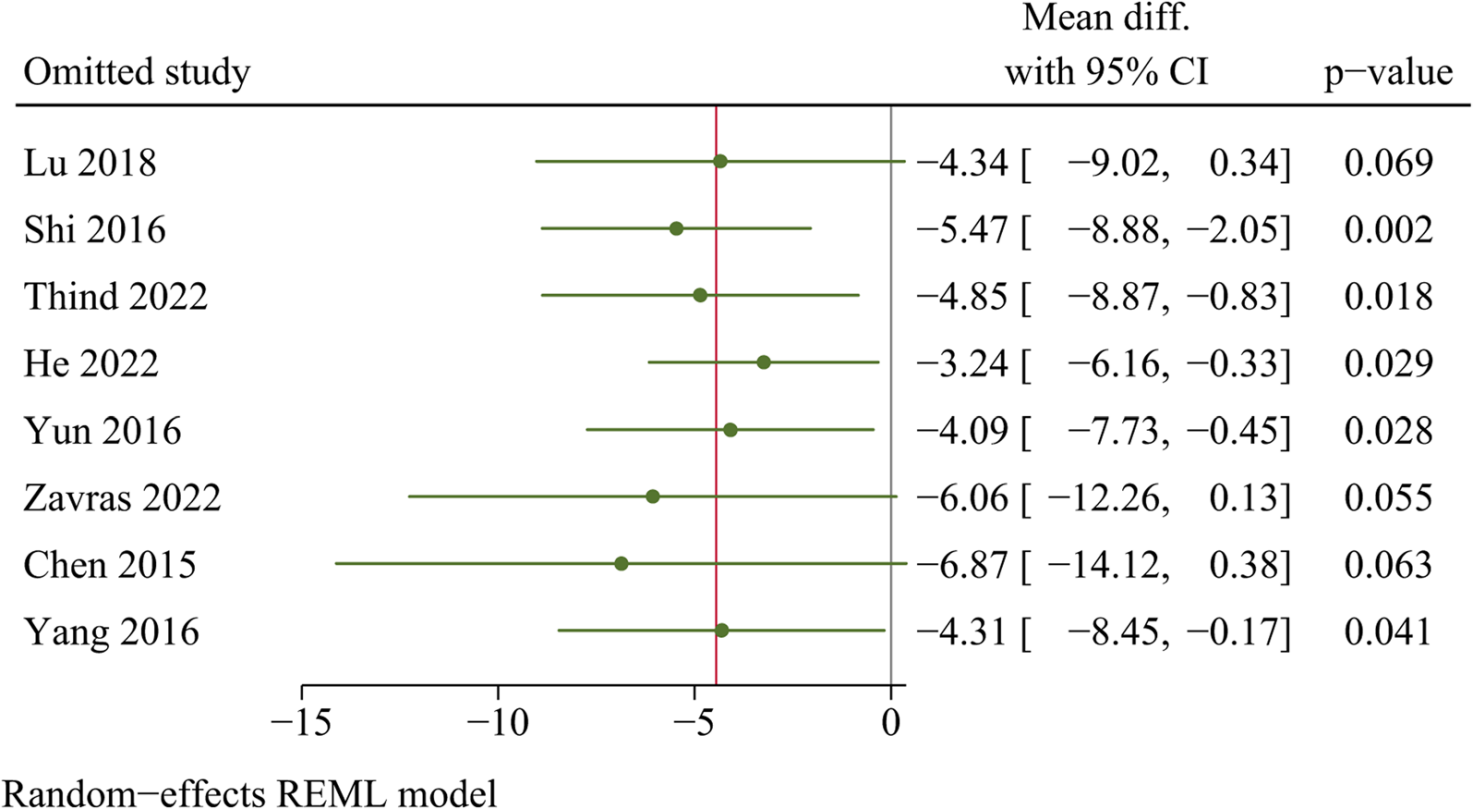
Sensitivity analysis of intraoperative bleeding

For sensitivity analysis, individual trials were sequentially excluded and then the remaining trials were combined. In the end, there was no statistically significant change in the effect sizes for the outcome indicators after excluding individual trials, except for intraoperative bleeding (Additional file 2). After excluding three studies separately, the pooled effect sizes for intraoperative bleeding changed significantly (Fig. 12). Consequently, the finding regarding intraoperative bleeding was not robust, and the pooled results for the remaining outcomes were robust.

### Subgroup analysis for possible causes of heterogeneity

Considerable heterogeneity existed between all included trials for surgical duration and intraoperative bleeding. Therefore, the sources of heterogeneity were further investigated by subgroup analysis. However, the results showed that the type of bilevel ACDF did not contribute to the heterogeneity.

### Publication bias assessment

Egger's test was applied for the evaluation of publication bias (Additional file 3). Eventually, the findings indicated no significant publication bias in any outcome (*p* > 0.05).

## Discussion

Nowadays, ACDF has become extensively accepted as the standard procedure for treating symptomatic monosegmental CDDD with satisfactory clinical outcomes. However, as the number of compressed segments increases, the pathological presentation becomes more complex, and currently the surgical decision remains controversial [9, 32–34]. Previously, numerous scholars undertook meta-analyses and compared the effectiveness and complications of ZAS and PCC in the treatment of single-level and multilevel CDDD, albeit with contradictory conclusions. Nambiar et al. concluded that ZAS minimised surgical duration, the incidence of postsurgical dysphagia in single-segment ACDF compared to PCC, although both implants provided similar effectiveness in restoring cervical curvature [35]. In contrast to their results, one meta-analysis conducted by Yang et al. demonstrated that ZAS was inferior to PCC in maintaining cervical flexion in multi-segmental ACDF, albeit with the superiority in diminishing the incidence of ASD after surgery [36]. In terms of postoperative cage subsidence, Lu et al. considered ZAS to have a higher risk than PCC, especially in multi-segmental ACDF [37].

Currently, the main surgical procedure for bilevel CDDD is still ACDF, in which the main intraoperative fixation and fusion devices applied are PCC and ZAS. Several clinical studies have compared the efficacy of both devices in the treatment of bilevel CDDD [22, 23, 26, 27]. However, there are few meta-analyses that evaluate the benefit and safety of ZAS versus PCC for bilevel ACDF. Our research was conducted to objectively assess the effectiveness and safeness of ZAS versus PCC in bilevel ACDF, with a goal of guiding clinicians on an evidence-based basis to select an appropriate implant in bilevel ACDF. Overall, our analysis demonstrated intraoperative bleeding and postoperative dysphagia rate to be significantly lower in ZAS group as compared to PCC group. However, there was no significant difference between both groups in operative time, JOA scores, NDI scores, cervical Cobb angle, fusion rates, ASD rates and cage sinking rates at the last follow-up.

As is well known, the unique integrated design of ZAS allows exposure of the pathological disc through a small incision, avoiding overexposure of the adjacent vertebral body. At the same time, the relatively simple implantation procedure significantly reduces surgical duration and intraoperative blood loss [32, 38, 39]. A meta-analysis found that ZAS in single-segment ACDF significantly reduced surgical duration and intraoperative bleeding than PCC [18]. Our study found that compared to PCC, ZAS significantly reduced intraoperative blood loss in bilevel ACDF, but there was no significant difference regarding operative time between both groups. In addition, there was considerable heterogeneity as regards operative time and intraoperative blood loss. We explored the source of heterogeneity by subgroup analysis, but the type of bilevel ACDF was not the cause of heterogeneity. Clinically, the duration of surgery and intraoperative blood loss are generally linked to the surgeon's operative experience and habits, as well as the patient's own condition, which may contribute to heterogeneity. It is worth noting that sensitivity analysis indicated the combined result of intraoperative bleeding was unrobust, so the result from the meta-analysis of intraoperative bleeding should be interpreted with caution.

Regarding the assessment of clinical outcomes in bilevel ACDF, JOA and NDI scores were similar in both groups at final follow-up. On the subgroup analysis, the results were also similar for both subgroups. This result suggested that the degree of symptom relief and neurological recovery was the same for ZAS and PCC in both continuous and noncontinuous bilevel ACDF. One potential rationale for this finding is as follows: while distinct fusion constructs are utilised, both surgical approaches directly decompress the spinal cord via removal of anterior pathological compression, for example, herniated discs, and hyperplastic bone at the posterior aspect of the vertebrae. A few trials found no significant differences between both interbody implants with regard to JOA score and NDI score at last follow-up for CDDD treatment [15, 34, 40]. Overall, our findings demonstrate both constructs have equally favourable effectiveness in both continuous and noncontinuous bilevel ACDF.

In terms of cervical Cobb angle and fusion rate at last follow-up, there was no significant difference between both groups for bilevel ACDF. Further subgroup analysis demonstrated no significant difference between both groups in either continuous or noncontinuous ACDF. Kahaer et al. carried out one meta-analysis and then their findings revealed no significant differences concerning cervical curvatures between ZAS and PCC at final follow-up [18]. Sun et al. observed similar fusion rate between the two groups at 5-year follow-up after three-segment ACDF [41]. Also, Abudouaini et al. reported no statistically significant difference in fusion rate between ZAS and PCC groups at last follow-up in single-segment ACDF [42]. ZAS may be inserted straight into the interbody spaces and screwed to the surrounding vertebral bodies via the endplates, thereby offering biomechanical stabilisation equivalent to an anterior plate and enhancing fusion rate. In addition, successful intervertebral fusion may prohibit loosening or subsidence of the fusion device, which may help decrease postsurgical reduction of cervical Cobb angle. Thus, the results suggest ZAS can obtain satisfactory fusion rates and maintain cervical curvature in the long term, the same as PCC in bilevel ACDF.

Dysphagia remains the most commonly reported complication of ACDF and is often attributed to the introduction of anterior plates and oesophageal traction intraoperatively. Studies showed ZAS significantly minimised the incidence of postoperative dysphagia in ACDF [32, 38, 43]. Our results demonstrated that the postoperative dysphagia rate was significantly smaller in ZAS group than in PCC group in bilevel ACDF. Further subgroup analysis indicated ZAS significantly diminished the incidence of postsurgical dysphagia in continuous bilevel ACDF, whereas there was no significant difference in the incidence of postsurgical dysphagia between the two groups in noncontinuous bilevel ACDF. ZAS can eliminate direct touch of the conventional plates against the oesophagus, thereby diminishing oesophageal irritation from the implant. Simultaneously, the placement of ZAS is more minimally invasive, reducing operative time and surgical field exposure, as well as reducing intraoperative stress on the prevertebral soft tissues and local soft tissue oedema during surgery. These advantages of ZAS decrease postoperative dysphagia rates in bilevel ACDF, particularly in continuous ACDF. Conversely, noncontinuous bilevel ACDF requires exposure of more surgical segments, greater surgical trauma and longer operative time, and undoubtedly increases prevertebral soft tissue oedema, which in turn promotes the development of postoperative dysphagia, offsetting the advantages of the ZAS in reducing the incidence of dysphagia. In conclusion, ZAS has an advantage over PCC in terms of reducing postoperative dysphagia in bilevel, especially continuous ACDF.

ZAS can avoid potential plate-induced irritation of adjacent segments, which is thought to be a predisposing factor for adjacent segment degeneration [44]. A biomechanical research suggested the presence of an anterior plate might increase loads on adjoining intervertebral discs and accelerate ASD [45]. Several studies showed that the incidence of postoperative ASD was significantly lower in the ZAS group than in the PCC group [38, 39, 46]. ZAS is capable of being implanted entirely within the interbody region, away from the adjacent levels. Conversely, the application of an anterior plate requires greater disruption and irritation of the soft tissues surrounding the adjacent segmental disc. The plate structure adds considerable stiffness and increases the stress on the adjacent segment, leading to a higher incidence of ASD. Meanwhile, micro-movement of the surgical segment reduces the stress on the adjacent segment and reduces the risk of ASD. With regard to ASD, our study found no significant difference between the two groups for either continuous or noncontinuous bilevel ACDF. In the future, randomised controlled trials with large samples, high quality and longitudinal follow-up are essential to better determine whether ZAS contributes to a reduction in the incidence of ASD.

Concerning cage subsidence, there was no significant difference between ZAS and PCC in bilevel ACDF. Our result is similar to the findings of He et al. and Zhao et al., which may be inferred from the similar biomechanical properties of the two devices and similar fusion rates [43, 47]. Overall, our findings suggested the adoption of ZAS in bilevel ACDF did not raise the incidence of cage sinking in comparison to PCC.

This article had a few merits. First of all, we developed rigorous study selection criteria. Only patients with clinically uncommon bilevel ACDF were included, hence minimising the expected biomechanical bias introduced by the count of discs across the entire analysis. Moreover, for further comparison of the effectiveness and security of ZAS versus PCC, we carried out subgroup analyses based on the type of bilevel ACDF and compared the clinical results of the two devices in the different types separately. Due to the rigorous inclusion criteria and exhaustive analysis in this research, possible confounding factors were minimised to enhance the validity and credibility of our findings. Furthermore, this analysis was subjected to Egger's test, which demonstrated that the potential for publishing bias was insignificant. Overall, this paper holds significant practical implications and is worthwhile to be interpreted with caution, thus offering clinicians more convincing guidance on the management of bilevel CDDD.

Some limitations of this research were likewise noted. First of all, due to the low natural incidence of bilevel CDDD, the sample size of included trials was small, consisting of just two randomised controlled trials. The selected articles were mainly observational, reducing the capacity of explaining the diverse clinical heterogeneity within ACDF and selection bias between ZAS and PCC groups. Furthermore, given the small number of trials, some clinical outcomes compared were not comprehensive. As there was no comprehensive analysis of spine alignment parameters such as C2-7 sagittal vertical axis and T1 slope, the results of this study might be underpowered to reflect changes in imaging outcomes. Also, because we focused only on two-segment ACDF, the results of this study did not necessarily apply to single-segment and multi-segment ACDF. Secondly, considerable heterogeneity between trials was observed among certain endpoint measures. Nevertheless, the subgroup analyses did not identify possible sources of heterogeneity, which could be related to the surgeon's clinical experience, surgical proficiency, the patient's own circumstances, etc. Thirdly, the type of ZAS was diverse across studies, which could be a source of bias in the findings. In addition, English was the language of all eligible trials. Hence, there could be a potential language bias. In the end, the unavailable medical costs in the trials involved in this review prevented a full comparison of the merits and demerits between both apparatuses. Consequently, given the shortcomings of our review, the above findings remain under validation by RCTs with large sample size, multicentre, longitudinal observation and high quality.

## Conclusion

Our findings demonstrated ZAS attained equivalent efficacy and security in bilevel ACDF compared to PCC with respect to operative time, JOA score, NDI score, cervical Cobb angle, fusion rates, cage sinking rates and ASD rates at final follow-up. Importantly, ZAS offered considerable superiority over conventional PCC concerning the reduction of intraoperative bleeding and postoperative dysphagia rates. Overall, ZAS is considered advantageous over PCC in bilevel ACDF. Given the limitations of our study, larger prospective randomised controlled trials are essential to generate further compelling evidence to consolidate our conclusions.

### Supplementary Information

**Additional file 1**: Search strategy.

**Additional file 2**: Sensitivity analysis.

**Additional file 3**: Publication bias assessment.

## Data Availability

Data backing up the results of this review are available in the article or its supplementary material. The protocol of this systematic review and meta-analysis is available in the Prospective Register of Systematic Reviews (PROSPERO), CRD 42023431169.

## Abbreviations

CDDD: Cervical degenerative disc disease
ACDF: Anterior cervical discectomy and fusion
PCC: Plate-cage construct
ASD: Adjacent segment degeneration
ZAS: Zero-profile anchored spacer
OS: Observational study
RCT: Randomized controlled trial
IBL: Intraoperative blood loss
JOA: Japanese Orthopaedic Association
NDI: Neck disability index
NOS: Newcastle–Ottawa Scale
OR: Odds ratios
CI: Confidence interval
WMD: Weighted mean difference
