# Supplementary material for: Zero-profile anchored spacer versus conventional plate-cage construct in bilevel anterior cervical discectomy and fusion: a systematic review and meta-analysis
Source: J Orthop Surg Res. 2023 Aug 31;18:644. doi: 10.1186/s13018-023-04134-4 (PMC10469803; doi:10.1186/s13018-023-04134-4)
Supplement: Supplementary file 1 — Additional file 1: Search strategy. [file 13018_2023_4134_MOESM1_ESM.pdf]

Search syntax of Pubmed, Web of Science and Cochrane Library:

(((((((((((zero profile) OR (zero-profile)) OR (zero-p)) OR (no-profile)) OR (Stand-alone)) OR (anchored spacer)) OR (anchored cage)) OR (Self-locking)) OR (ROI-C)) OR (Prevail)) AND ((anterior cervical discectomy and fusion) OR (ACDF))) AND (plate)

Search syntax of Embase:

Embase session results (1 Jun 2023)

No.	Query	Results
#4	#1 AND #2 AND #3	263
#3	plate	190628
#2	('anterior cervical discectomy'/exp OR 'anterior cervical discectomy' OR (anterior AND cervical AND ('discectomy'/exp OR discectomy))) AND ('fusion'/exp OR fusion) OR acdf	5860
#1	zero AND ('profile'/exp OR profile) OR 'zero profile' OR 'no profile' OR 'zero p' OR 'stand alone' OR (anchored AND spacer) OR (anchored AND cage) OR 'self locking' OR 'roi c' OR prevail	27774
