# Supplementary material for: Zero-profile anchored spacer versus conventional plate-cage construct in bilevel anterior cervical discectomy and fusion: a systematic review and meta-analysis
Source: J Orthop Surg Res. 2023 Aug 31;18:644. doi: 10.1186/s13018-023-04134-4 (PMC10469803; doi:10.1186/s13018-023-04134-4)
Supplement: Supplementary file 2 — Additional file 2: Sensitivity analysis. [file 13018_2023_4134_MOESM2_ESM.pdf]

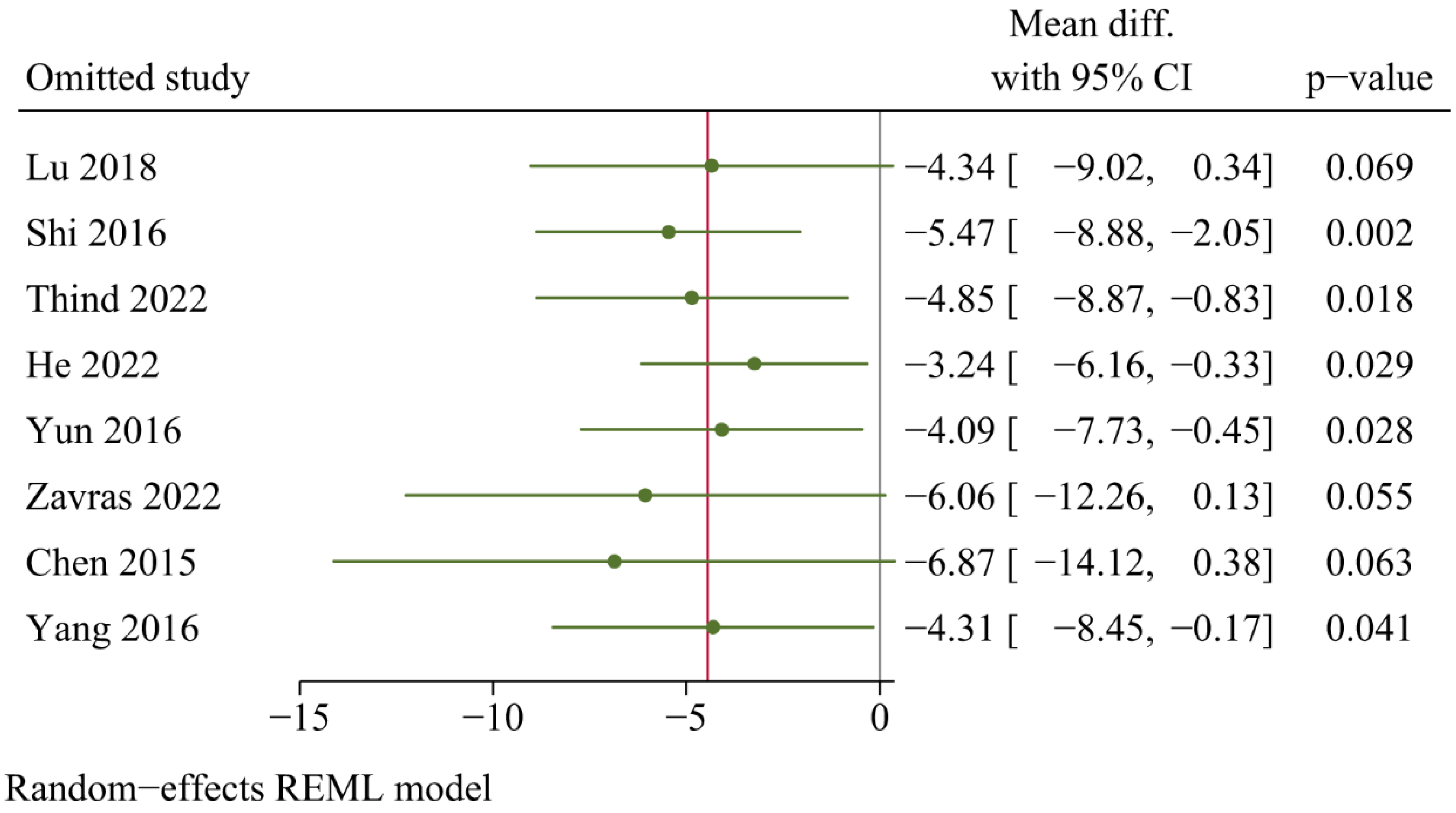

Sensitivity analysis for intraoperative blood loss

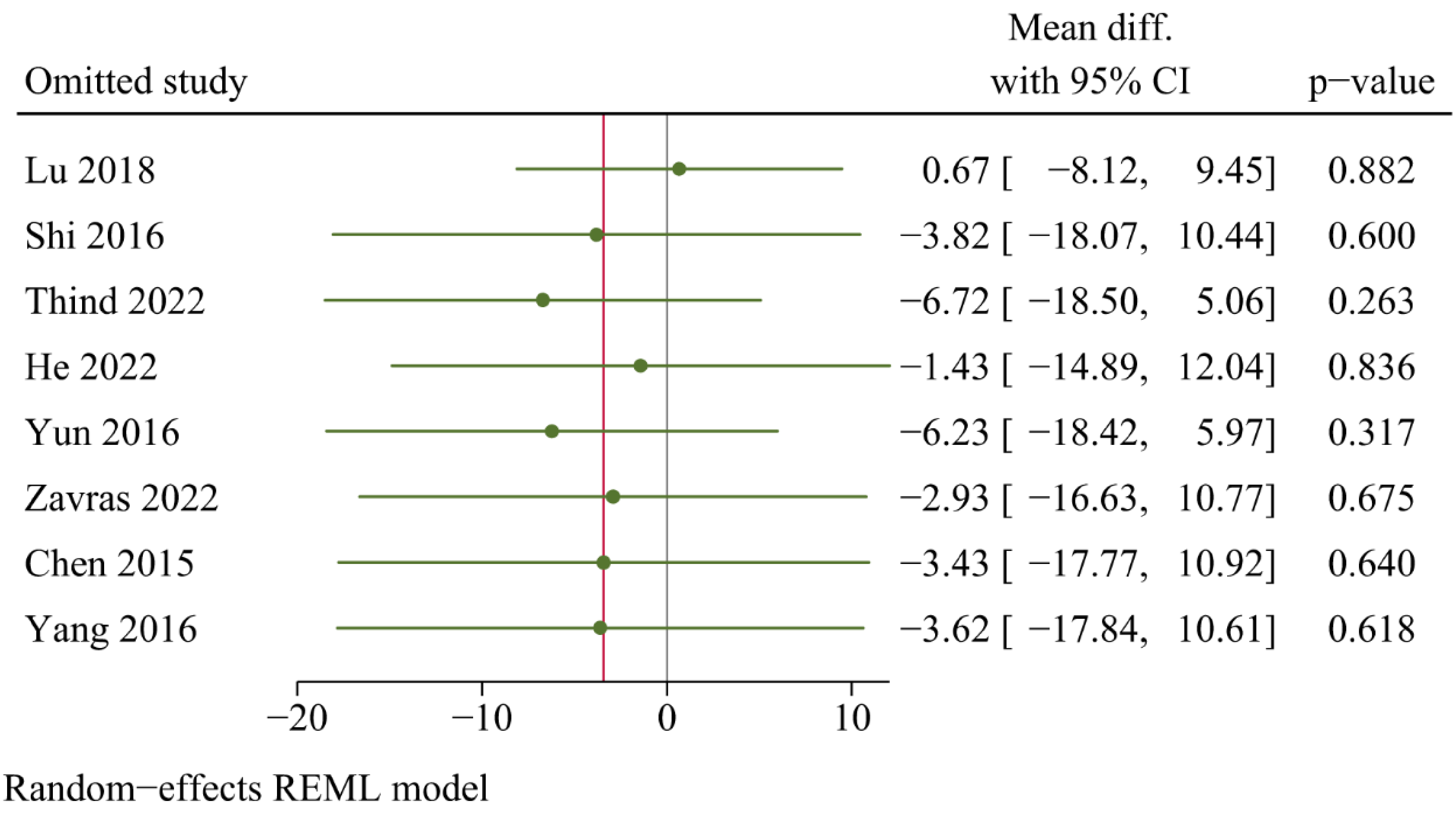

Sensitivity analysis for operation time

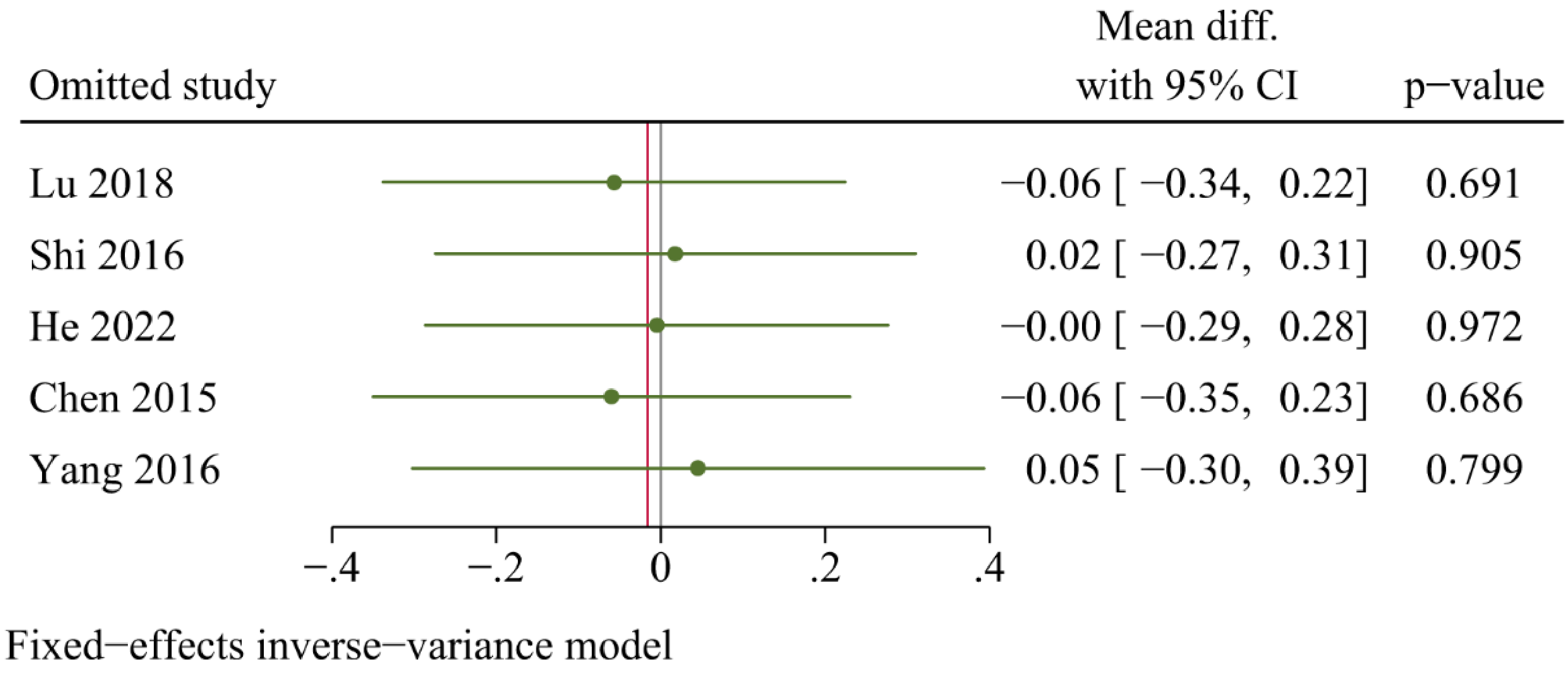

Sensitivity analysis for JOA scores at final follow-up

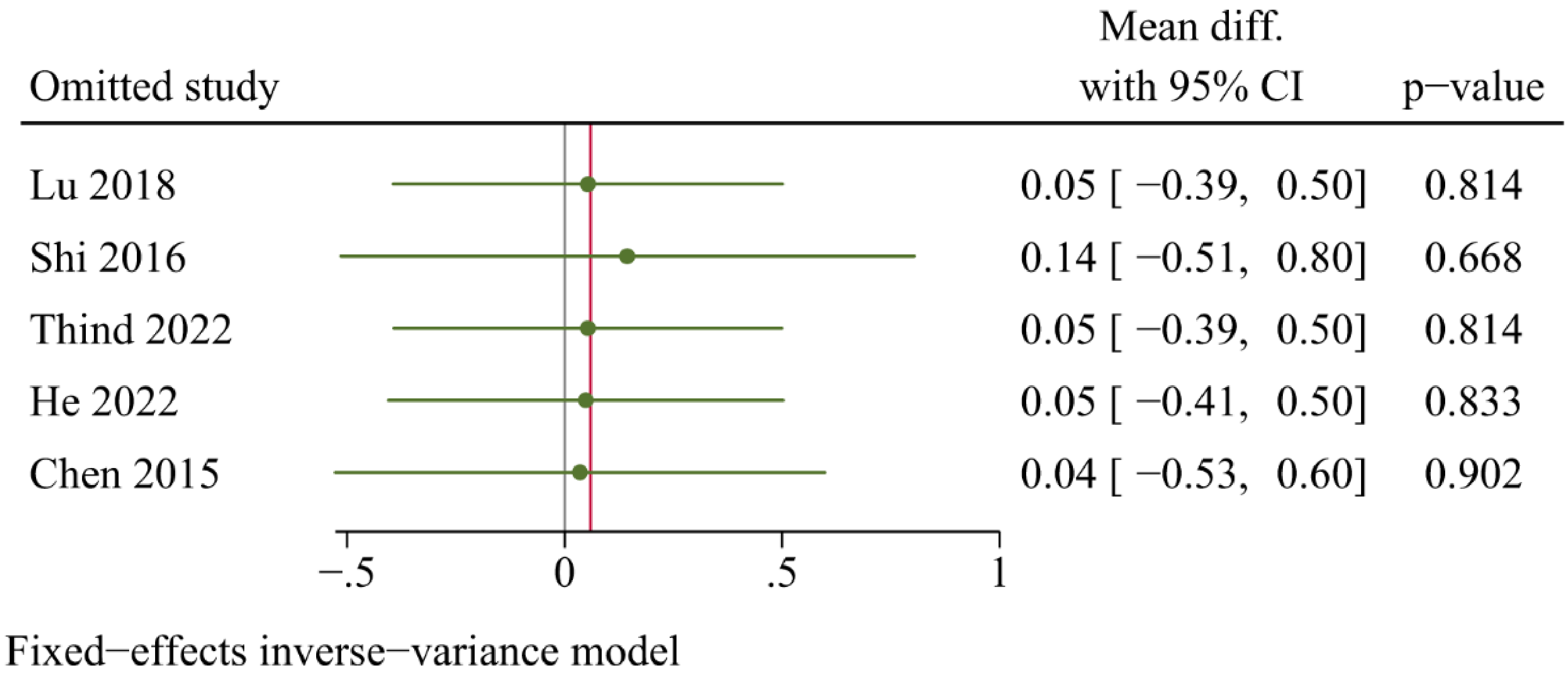

Sensitivity analysis for NDI scores at final follow-up

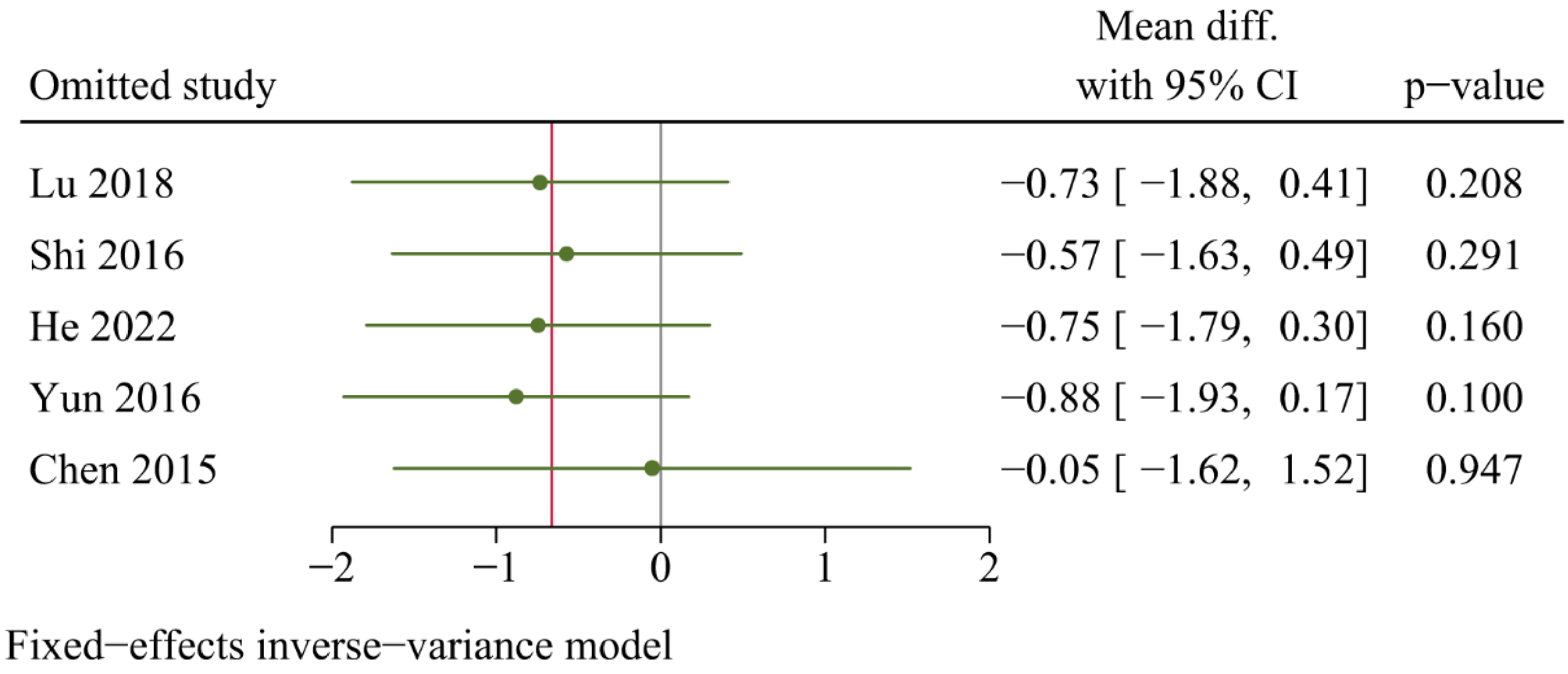

Sensitivity analysis for cervical Cobb angle at final follow-up

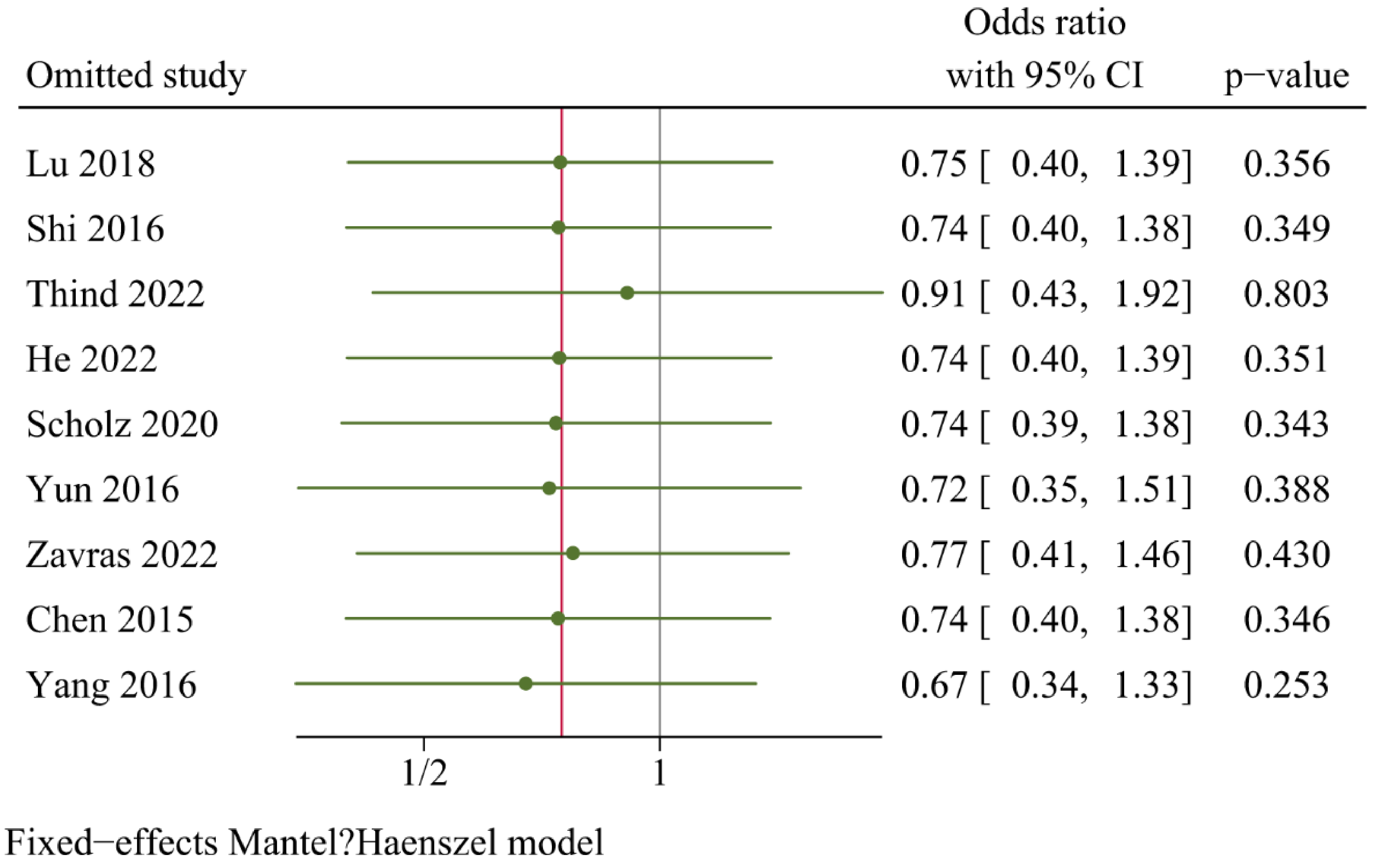

Sensitivity analysis for fusion rate

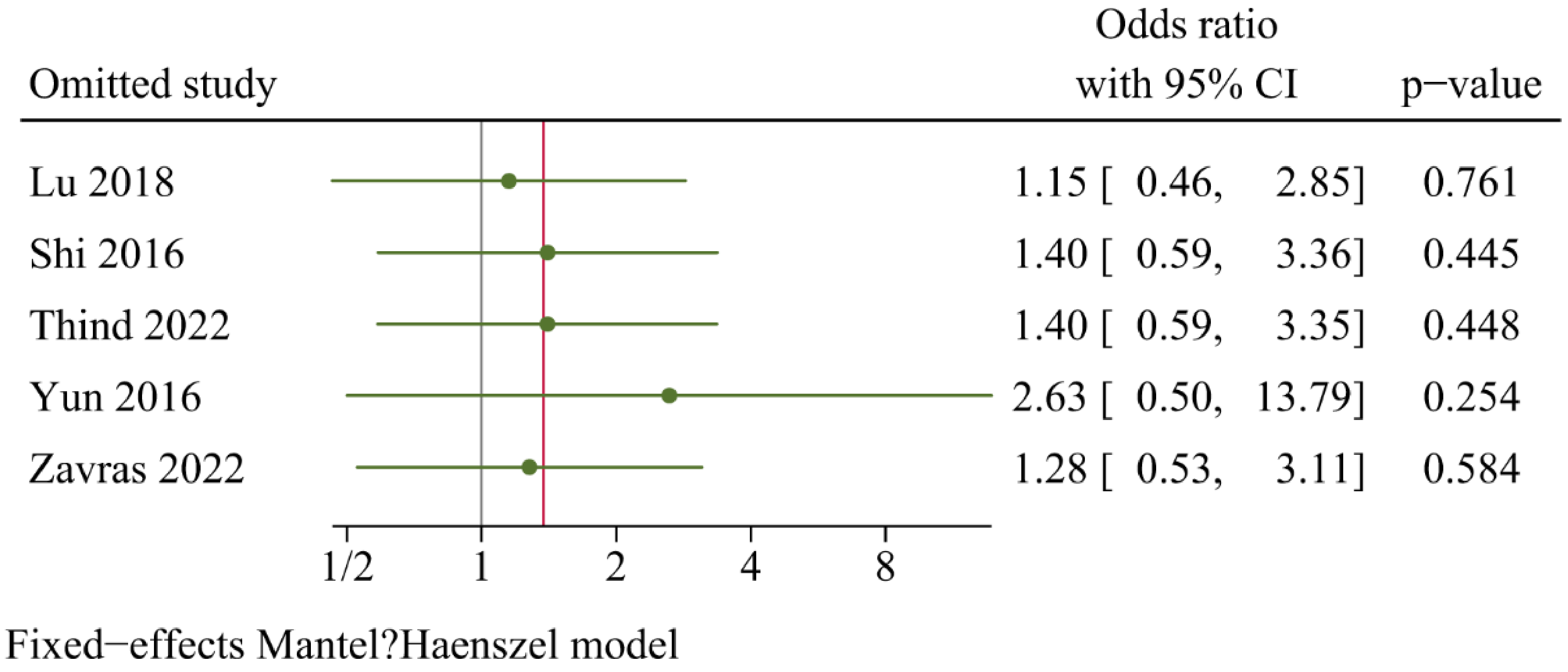

Sensitivity analysis for cage subsidence rate

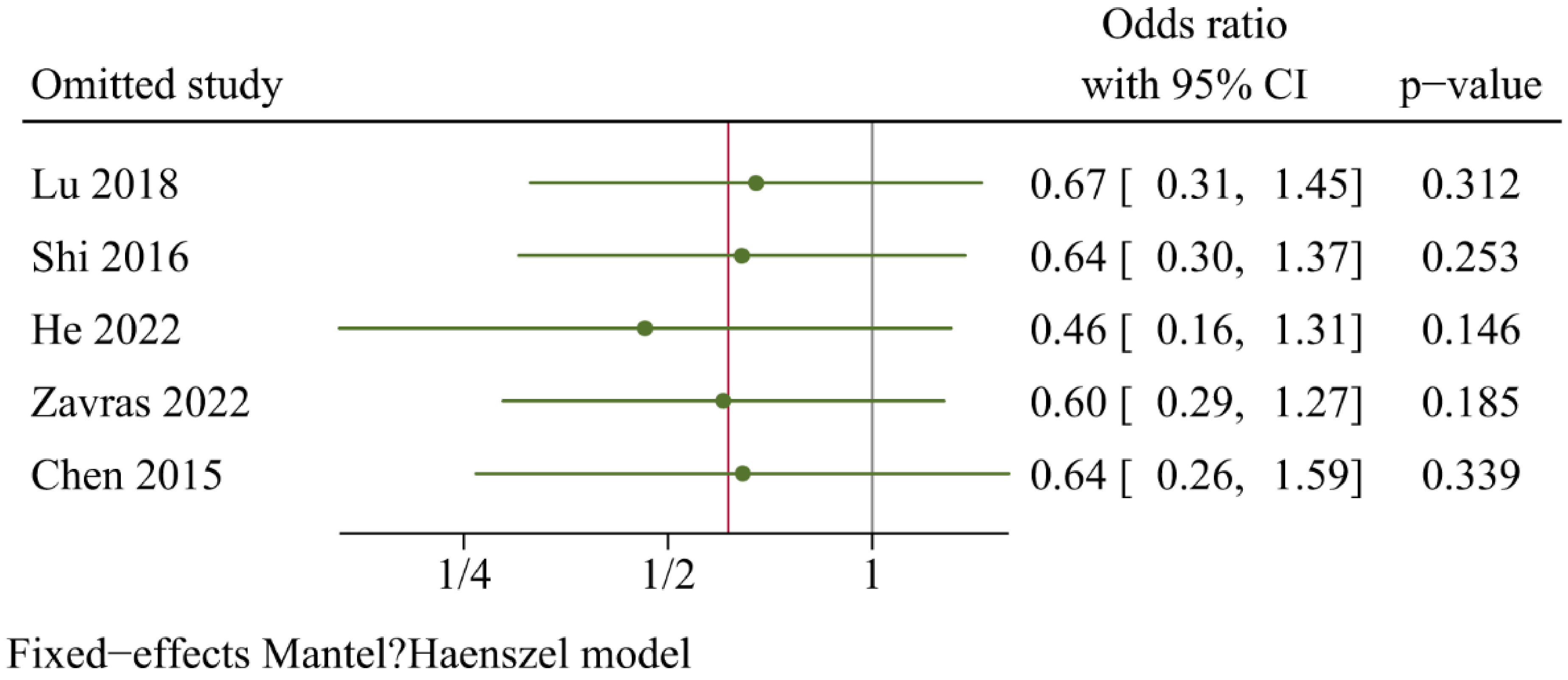

Sensitivity analysis for adjacent segment degeneration rate

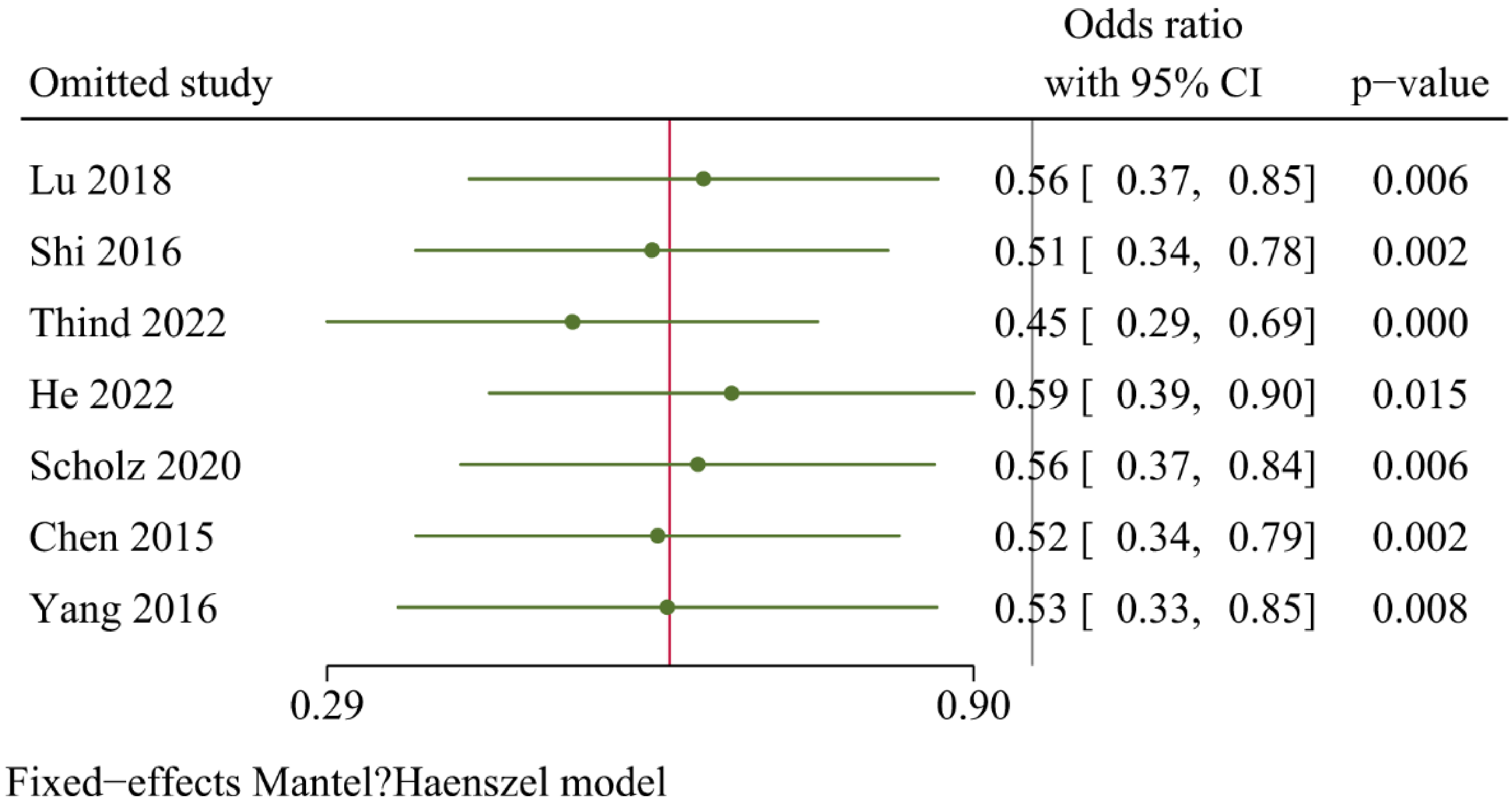

Sensitivity analysis for postoperative dysphagia
