# Supplementary material for: Zero-profile anchored spacer versus conventional plate-cage construct in bilevel anterior cervical discectomy and fusion: a systematic review and meta-analysis
Source: J Orthop Surg Res. 2023 Aug 31;18:644. doi: 10.1186/s13018-023-04134-4 (PMC10469803; doi:10.1186/s13018-023-04134-4)
Supplement: Supplementary file 3 — Additional file 3: Publication bias assessment. [file 13018_2023_4134_MOESM3_ESM.pdf]

```
. meta bias, egger

  Effect-size label: Mean diff.
        Effect size: _meta_es
          Std. err.: _meta_se

Regression-based Egger test for small-study effects
Random-effects model
Method: REML

H0: beta1 = 0; no small-study effects
            beta1 =      -1.14
      SE of beta1 =      0.647
                z =      -1.76
       Prob > |z| =     0.0787

.
```

Publication bias assessment of intraoperative blood loss

```
. meta bias, egger

  Effect-size label: Mean diff.
        Effect size: _meta_es
          Std. err.: _meta_se

Regression-based Egger test for small-study effects
Random-effects model
Method: REML

H0: beta1 = 0; no small-study effects
            beta1 =       1.85
      SE of beta1 =      1.969
                z =       0.94
       Prob > |z| =     0.3475

.
```

Publication bias assessment of operation time

```
. meta bias, egger

  Effect-size label: Mean diff.
        Effect size: _meta_es
          Std. err.: _meta_se

Regression-based Egger test for small-study effects
Fixed-effects model
Method: Inverse-variance

H0: beta1 = 0; no small-study effects
            beta1 =      1.09
      SE of beta1 =     1.756
                z =      0.62
       Prob > |z| =    0.5367

.
```

Publication bias assessment of JOA scores at the final follow-up

```
. meta bias, egger

  Effect-size label: Mean diff.
        Effect size: _meta_es
          Std. err.: _meta_se

Regression-based Egger test for small-study effects
Fixed-effects model
Method: Inverse-variance

H0: beta1 = 0; no small-study effects
            beta1 =      0.32
      SE of beta1 =     0.760
                z =      0.43
       Prob > |z| =    0.6691

.
```

Publication bias assessment of NDI scores at the final follow-up

```
. meta bias, egger

  Effect-size label: Mean diff.
        Effect size: _meta_es
          Std. err.: _meta_se

Regression-based Egger test for small-study effects
Fixed-effects model
Method: Inverse-variance

H0: beta1 = 0; no small-study effects
            beta1 =      1.26
      SE of beta1 =     1.011
                z =      1.25
       Prob > |z| =    0.2116

.
```

Publication bias assessment of cervical Cobb angle at final follow-up

```
. meta bias, egger
note: declared Mantel-Haenszel method not supported with meta bias; using inverse-variance method

  Effect-size label: Log odds-ratio
        Effect size: _meta_es
          Std. err.: _meta_se

Regression-based Egger test for small-study effects
Fixed-effects model
Method: Inverse-variance

H0: beta1 = 0; no small-study effects
            beta1 =      0.27
      SE of beta1 =     0.683
                z =      0.39
       Prob > |z| =    0.6931

.
```

Publication bias assessment of fusion rate

```
. meta bias, egger
note: declared Mantel-Haenszel method not supported with meta bias; using inverse-variance method

  Effect-size label: Log odds-ratio
        Effect size: _meta_es
          Std. err.: _meta_se

Regression-based Egger test for small-study effects
Fixed-effects model
Method: Inverse-variance

H0: beta1 = 0; no small-study effects
            beta1 =      0.52
      SE of beta1 =     0.807
                z =      0.64
       Prob > |z| =    0.5234

.
```

Publication bias assessment of cage subsidence rate

```
. meta bias, egger
note: declared Mantel-Haenszel method not supported with meta bias; using inverse-variance method

  Effect-size label: Log odds-ratio
        Effect size: _meta_es
          Std. err.: _meta_se

Regression-based Egger test for small-study effects
Fixed-effects model
Method: Inverse-variance

H0: beta1 = 0; no small-study effects
            beta1 =     -0.57
      SE of beta1 =     0.931
                z =     -0.61
       Prob > |z| =    0.5401

.
```

Publication bias assessment of adjacent segment degeneration rate

```
. meta bias, egger
note: declared Mantel–Haenszel method not supported with meta bias; using inverse-variance method

  Effect-size label: Log odds-ratio
        Effect size: _meta_es
          Std. err.: _meta_se

Regression-based Egger test for small-study effects
Fixed-effects model
Method: Inverse-variance

H0: beta1 = 0; no small-study effects
            beta1 =     -0.87
      SE of beta1 =     1.643
                z =     -0.53
       Prob > |z| =    0.5957

.
```

Publication bias assessment of postoperative dysphagia rate
